# Advances in functional coatings on biliary stents

**DOI:** 10.1093/rb/rbae001

**Published:** 2024-01-18

**Authors:** Kaining Yang, Wenxin Sun, Lanyue Cui, Yuhong Zou, Cuie Wen, Rongchang Zeng

**Affiliations:** Department of Bioengineering, College of Chemical and Biological Engineering, Shandong University of Science and Technology, Qingdao 266590, China; Department of Bioengineering, College of Chemical and Biological Engineering, Shandong University of Science and Technology, Qingdao 266590, China; Corrosion Laboratory for Light Metals, College of Materials Science and Engineering, Shandong University of Science and Technology, Qingdao 266590, China; Department of Bioengineering, College of Chemical and Biological Engineering, Shandong University of Science and Technology, Qingdao 266590, China; School of Engineering, RMIT University, Melbourne, VIC 3001, Australia; Corrosion Laboratory for Light Metals, College of Materials Science and Engineering, Shandong University of Science and Technology, Qingdao 266590, China

**Keywords:** biliary stent, coating, anticorrosion, antibacterial, antitumor, stone-dissolving, X-ray visibility, antistent migration

## Abstract

Biliary stenting is an important interventional method for the prevention and treatment of biliary tract diseases. However, complications, such as postoperative biliary infection and restenosis, frequently occur due to the extensive scope of the biliary system and the complex composition of bile. The combination of coating technology and biliary stents is expected to bring new approaches to the solution of these problems. The cutting-edge advance on functional coatings on biliary stents is reviewed from seven perspectives: anticorrosion, -bacterial, -tumor, stone-dissolving, X-ray visibility, antistent migration and functional composite coatings. The development trend is also discussed. Overall, the performance of the numerous functional coatings for various purposes is generally up to expectations, but the balance between the medications’ effectiveness and their safety needs to be further adjusted. Many contemporary investigations have advanced to the level of animal experiments, offering crucial fundamental assurance for broader human studies. The combination of biliary stents and functional coatings is an innovative idea with great potential for future development.

## Graphical Abstract

**Figure:**
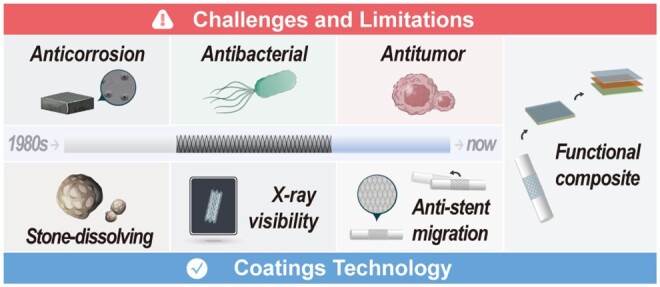

## Introduction

**Figure 1.**
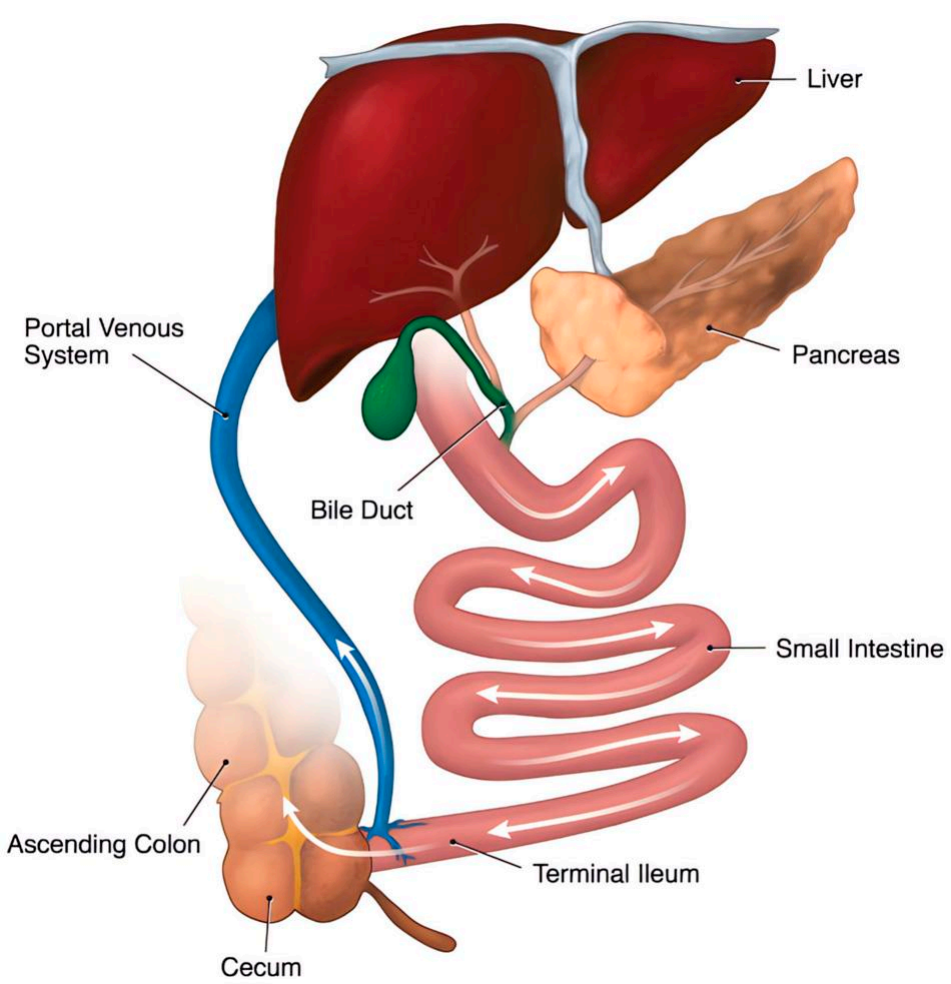
Enterohepatic circulation. Reproduced from Ahmed *et al.* [2] with permission from Copyright 2022 Dove Medical Press.

The biliary tract is made up of the gallbladder, common hepatic duct and common bile duct. It starts inside the liver and finishes in the descending duodenum and controls secreting, storing, concentrating and transporting of bile. The liver produces bile, which is subsequently collected and discharged into the gallbladder and the second half of the duodenum. When bile reaches the end of the ileum, it is absorbed into the portal circulation through the terminal mucosa of the ileum and then travels back to the liver to be absorbed by hepatocytes. This process is known as enterohepatic circulation [1]. Non-steroidal anti-inflammatory drugs, antibiotics and other drugs and hormones as well are secreted from liver cells into bile and enterohepatic circulation; many lipophilic exogenous drugs can also exploit the enterohepatic circulation and cause liver damage. Figure 1 shows enterohepatic circulation [2].

Inflammation (inflammatory strictures, cholangitis), stones (gallbladder stones, common hepatic duct stones and hepatobiliary duct stones), and malignancies (bile-duct cancer and bile bladder cancer) are some of the disorders that affect the biliary tract. Between 1990 and 2019, the global and regional incidence of gallbladder and biliary tract diseases (GBTDs) increased rapidly from 26.35 to 52 million cases per year; the age-standardized incidence rose from 585.35 to 634.32 per 100 000 [3]. As a result, GBTDs have emerged as an important threat to human health that require constant monitoring and attention.

Surgery is the standard treatment for GBTDs. In 1980, Soehendra *et al.* [4] first applied plastic biliary stents for the treatment of biliary stenosis, laying the foundation for large-scale clinical applications of biliary stents. Biliary stents are currently the method of choice for the improvement of benign biliary strictures and an effective tool for the palliative treatment of malignant biliary tract disease. Biliary stenting often requires specialized techniques, such as endoscopic retrograde cholangiopancreatography (ERCP) or percutaneous transhepatic cholangial drainage, for the successful implantation of a stent into a specific site, but it has significant advantages over conventional surgical treatment, including simplicity of operation, less trauma and invasiveness and fewer complications.

In more than 40 years since biliary stents were first created and implemented, numerous stent substrate materials, structural designs and manufacturing techniques have been developed in response to the shifting demands of medicine. In this review, we provide a summary of the history of biliary stent development, with a focus on recent advancements in biliary stent coating technologies and future development trends.

## Development of biliary stents

The original biliary stents developed in the 1980s were mainly composed of non-biodegradable plastics. Self-expanding metal stents (SEMSs), which have advantages in prolonging stent patency time [5], were introduced in 1989. SEMSs can expand to their normal size, can provide longer stent opening time [6] due to their larger diameter and higher mechanical tension and can easily be manipulated once inserted. Bioresorbable stents are the most recent biliary stent technology developed to resolve issues of difficult stent removal and increased risk of complications from repeated surgery due to tissue overgrowth, the stent substrate is made of corrosive metal or degradable polymers that degrades into a natural byproduct after performing its intended support function [7, 8]. At present, there is no biodegradable biliary stent approved by the US Food and Drug Administration. In 2021, a balloon expandable biodegradable biliary stent called UNITY-B (Q3 Medical Devices) obtained the CE marking of European Union. The fully biodegradable stent is designed with a ‘musculoskeletal system’ as the design idea. Magnesium alloy substrate simulates bone as the main support structure, and polymer coating simulates muscle to help support movement and stability. The new biliary stent has been used in postcholecystectomy perihilar benign biliary stricture on the clinic [9]. The comparison of polymeric and metallic biliary stents is listed in Table 1 and a detailed comparison of different types of SEMSs is shown in Table 2.

**Table 1.** Comparison of polymeric and metallic biliary stents^a^

		Polymer stents		Metal stents	
		Degradable	Non-degradable	Degradable	Non-degradable
Materials		Polylactide (PLA), polyglycolide (PGA), polycaprolactone (PCL), polydioxanone (PDO or PDX)	Polyethylene (PE), polyurethane (PU), polytetrafluoroethylene (PTFE)	Magnesium (Mg), zinc (Zn), iron (Fe) and their alloys	Stainless steel, nickel–titanium alloys (nitinol)
Advantages		Spontaneous degradation, no need for secondary surgery to remove	Low cost, easy to place and remove, available in many shapes and sizes	Spontaneous degradation, no need for secondary surgery to remove	Large lumen diameter, long patency time, strong support force
Disadvantages		Potential for stent occlusion and migration, some materials have weak supporting force	Short patency period, easily occlude again, weak support force, risk of migration	Potential for stent occlusion and migration, unsuitable degradation rates for some metals	Difficult to remove or replace
Approved biliary stent	Product	ARCHIMEDES ™ biodegradable biliary and pancreatic stent	Advanix ™ Biliary stent	UNITY-B ™ Percutaneous balloon expandable biodegradable biliary stent	WallFlex ™ Biliary RX Stent
	Materials	Polydioxanone, polyethylene glycol and barium sulfate (*) Polydioxanone and barium sulfate (**) Poly (lactide-co-caprolactone-co-trimethylene carbonate) and barium sulfate (***)	Polypropylene (20% BiOCl)	MgNdMn21 alloy	Nitinol and platinum
	Design	Outside spiral	Thin wall design, tapered stent tip	Large diameter, expandable	Closed-cell, braided design, looped and flared stent end

a Three kinds of Archimedean stents with different degradation rates were designed by using different materials, namely

* Fast,

** Medium and

*** Slow.

**Table 2.** Strengths and disadvantages of different types of self-expandable metal stents^a^

	Uncovered self-expandable metal stents	Partially covered self-expandable metal stents	Fully covered self-expandable metal stents
Strengths	Long stent patency due to large luminal diameter; stent malposition is rare	Long stent patency due to large luminal diameter; biliary leakage is rare	Long stent patency due to large luminal diameter, covering inhibits tissue/tumor in-growth through mesh; no biliary leakage
Disadvantages	Cost-effective for use >4 months; high risk for tissue ingrowth or tumor overgrowth; risk for duodenal wall erosion; biliary leakage possible; difficult to reposition or remove	Cost-effective for use >4 months; intermediate risk for tissue ingrowth or tumor overgrowth; risk for duodenal wall erosion; difficult to reposition or remove; side branch obstruction possible	Cost-effective for use >4 months; risk for duodenal wall erosion; difficult to reposition or remove; high risk of stent migration; side branch obstruction possible

a Reproduced from Lam *et al.* [23] with permission from Copyright 2021 Baishideng Publishing Group Inc.

With the ongoing advancement of material science, the structural design of biliary stents has also been improved to significantly enhance stent performance and broaden the usage scenario. Kwon *et al.* [10] have been developed stents with anchor flaps that prevent stent migration; Cho *et al.* [11] developed a flexible helical spring structure to increase the compliance of the stent and bile duct structure. Furthermore, the production of stents has kept up with the advancement of contemporary technology and some contemporary manufacturing methods have enhanced the efficiency and quality of biliary stent production. 3D printing (3DP) is an emerging manufacturing technology, it has been widely used in bone materials [12–14], oral materials [15–17], vascular materials [18–20], etc. Park *et al.* [21] used magnetic resonance imaging and 3DP along with a series of other processes to successfully prepare ultrathin tubular freeform structures providing sufficient mechanical flexibility for bile duct reconstruction in rabbits. The combination of 3DP technology and smart materials can provide new solutions for self-expanding supports with shape-memory characteristics [22].

## Functional coatings of biliary stents

Biliary stents have become more widely used, but there have been increases in complications, such as post-implantation infection and restenosis. Improvement of biliary stent performance through surface-modification techniques, loading of radioactive particles [24] and other methods has been considered, with the coating being a more mature surface treatment and currently the main research direction of surface-modification techniques for biliary stents. Coatings are thin layers of materials that improve the surface properties of a substrate and create a protective barrier against harmful external factors when deposited or applied to its surface [25]. Coating technology plays an important role in the preparation of drug-eluting stents, promising targeted improvements in stent performance based on specific structural designs and suitable substrates.

### Anticorrosion coatings

Bioabsorbable stents are mainly made of polymer materials or biodegradable metals. They can compensate for the inadequacies of conventional plastic and metal stents, and can also be employed as drug-delivery vehicles for disease treatment or to inhibit endothelial growth [26].

Mg and its alloys have so good biocompatibility that the Mg^2+^ released by it has been shown to have certain anti-inflammatory [27], and the degradation products Mg^2+^ and OH^−^ also have inhibitory effects on gallbladder cancer cells [28], which is a suitable substrate for the construction of biliary stents. However, in many scenarios the basic structure of Mg alloy stents changes before they reach the end of their intended service period and thus, they lose their normal function. The *in vivo* corrosion of Mg–6Zn alloy-based biodegradable common bile duct stents was assessed and the residual weight of the Mg–6Zn alloy was determined to be only 9% of the original weight 3 weeks after implantation [29]. It shows that it is imperative to decrease the corrosion rate of Mg–6Zn alloy stents.

The quick degradation of Mg implants is a significant obstacle to broader clinical implementation [30], coating technology is the most common technical means of improving corrosion resistance. Chiu *et al.* [31] bathed pure Mg samples in 48% hydrofluoric acid for 24 h at room temperature to generate a homogeneous dense coating with a thickness of ∼1.5 μm. After testing in Hanks’ solution, the fluoride-coated sample experienced a far less severe and more uniform corrosion compared to the untreated sample.

**Figure 2.**
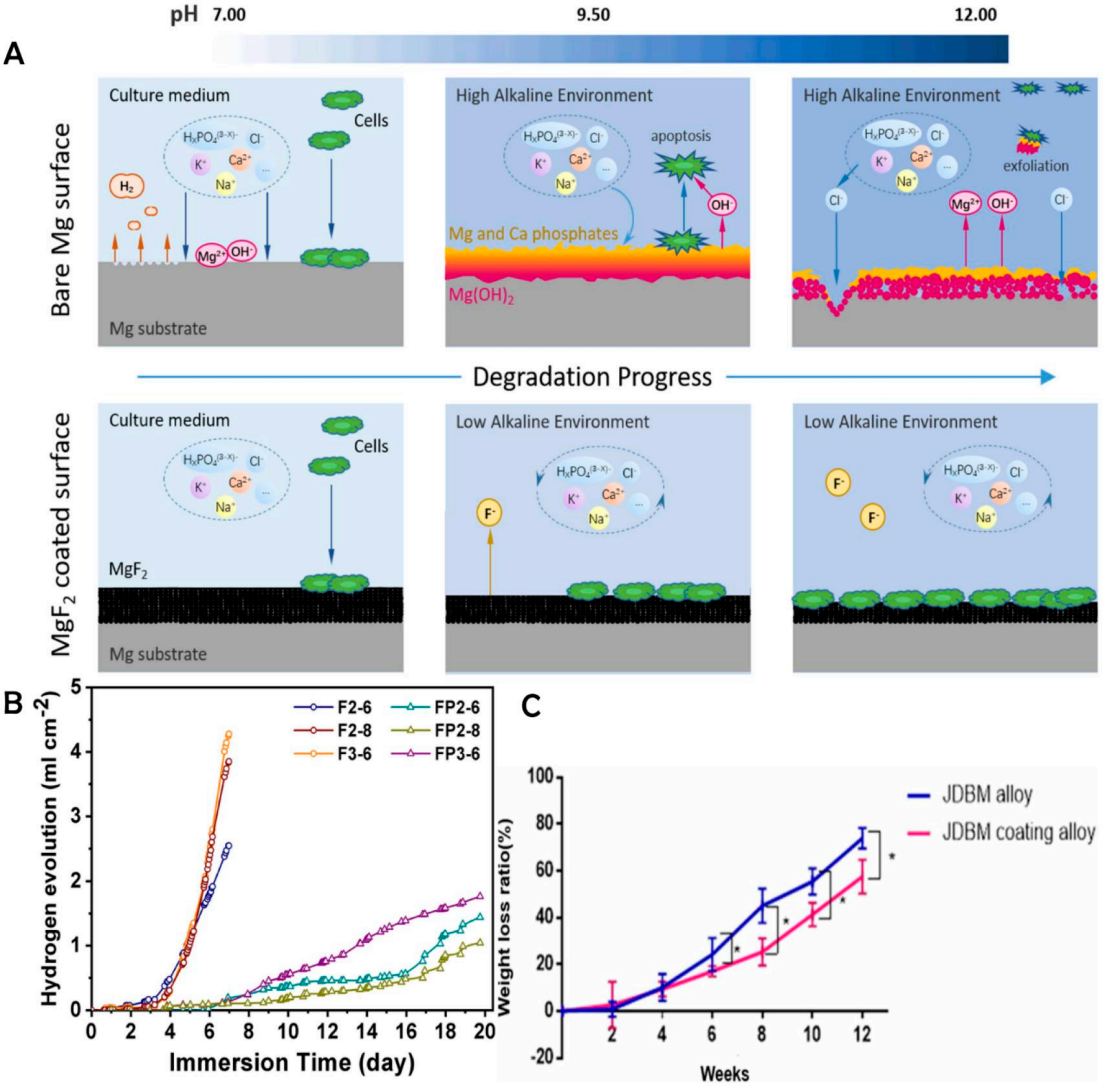
(**A**) Schematic illustration of degradation mechanism and biological properties of bare and MgF_2_-coated Mg; reproduced from Lou *et al.* [33] with permission from Copyright 2021 Elsevier B.V. (**B**) Evolution of hydrogen in simulated bile at 37°C for different samples; reproduced from Zhang *et al.* [34] with permission from Copyright 2020 Springer Nature. (**C**) Weight loss ratio of JDBM alloy stent group and JDBM coating stent group (∗represents *P* < 0.05). Reproduced with permission from Guo *et al*. [37].

Tian *et al.* [32] made coating containing fluorine on AZ31 alloy surface by plasma electrolytic oxidation technology and found that the addition of fluorine had no obvious toxic effect on cells, while enhancing cell adhesion on the surface of the coating and corrosion resistance of the coating *in vivo* and *in vitro*. Lou *et al.* [33] proposed the degradation mechanism of pure Mg and MgF_2_ coated in a dielectric environment: the environment’s pH was significantly raised at the beginning of the process as a result of the pure Mg surface reacting with the medium to produce compounds like Mg(OH)_2_, which caused the majority of cells to undergo apoptosis and die. The Mg(OH)_2_ corrosion product film was then gradually disintegrated by the Cl^−^ ions in the medium, creating a loose porous structure that partially detached the Mg(OH)_2_ membrane and aided in the continued deterioration of the Mg matrix. The dense and stable surface of the MgF_2_ film provides a suitable environment for cell adhesion at the initial stage and the environmental pH does not suddenly increase due to the protection of the Mg substrate by the MgF_2_ film; then it gradually thins out due to the decomposition of the F^−^ in the MgF_2_ film into the medium, but there are no corrosion craters on the surface, which is a structural factor in the enhancement of cytocompatibility. The above process is shown in Figure 2A. Additionally, the fluorinated product MgF_2_ is particularly well suited for biomaterials because it is easy to prepare, adheres to the substrate well and does not significantly alter material dimensions. It is also anticipated to significantly enhance the overall performance of the stent.

Zhang *et al.* [34] applied two types of coatings to the biodegradable ZX20 alloy biliary stent: fluorinated stents without PCL loading, and PCL-coated stents. They compared the long-term corrosion resistance of the two coatings in simulated bile. The rate at which the Mg alloy corroded was positively related to the amount of hydrogen released. Both surface treatments were successful in preventing corrosion at the beginning of immersion as evidenced by the small number of bubbles that gathered on the surfaces of the fluorinated stents without PCL loading (F2–6, F2–8 and F3–6) and the absence of bubbles on the surfaces of the PCL-coated samples (FP2–6, FP2–8 and FP3–6). However, after 4 days of immersion the hydrogen volume of the fluorinated non-coated sample increased quickly, whereas that of the PCL-coated stents began to increase gradually after 6 days of immersion and accelerated after 13–15 days, as shown in Figure 2B. The corrosion on the non-coated stent was already severe on Day 6 of immersion, with the appearance of structural fracturing. In contrast, the PCL-coated stent remained intact after 8 days of immersion with only minute corrosion pits on the surface, while dense corrosion pits appeared after 15 days. All stents degraded preferentially toward their ends; however, for the uncoated stents corrosion was also likely to occur near the intersection of the wire mesh. According to the above, the PCL coating considerably increased stent corrosion resistance.

Zhang *et al.* [35] and Shi *et al.* [36] prepared an Mg–Nd–Zn–Zr alloy (JDBM). Guo *et al.* [37] established a polylactic acid (PDLLA) coating on the surface of fluoride-treated JDBM to create a protective layer called MgF_2_/PDLLA. The purpose of this protective layer is to mitigate the quick degradation of the bare alloy. Two different types of biliary stents (JDBM stent and the coating JDBM stent) were inserted into a bile perfusion device for corrosion testing. As can be seen, during the first 6 weeks of immersion the two stents’ degradation rates were fairly slow and their weight loss rates were similar, after that the two stents’ corrosion rates were noticeably higher and the mass of the bare JDBM stent decreased significantly more quickly than that of the coating JDBM stent (Figure 2C). After 8 weeks, corrosion pits occurred on both the coated JDBM stents and bare stents. The corrosion of the bare stents was more severe. After 12 weeks, the structure of the bare stent was completely damaged and lost its original function. However, the coated JDBM stent maintains its fundamental structure. The JDBM stents and the coated JDBM stents were implanted into the same number of beagles, all stent structures were still intact after 15 days but had mostly disappeared by Day 30, But the coated JDBM stent group contained more leftover Mg alloy wire than the bare stent group. It took 60 days for all the stents to completely degrade. Therefore, the coated JDBM stents had reduced corrosion rates and improved corrosion resistance compared to the bare stents. In addition, through *in vitro* cell tests and *in vivo* experiments on animals, these stents have been proved to be biosafe to L-929 cells and have good biocompatibility to experimental animals.

Mg can create passivation layers in some bodily fluids [38], which can reduce the rate of substrate corrosion. However, the composition of bile is more complex and there are variations in the compositions of human and animal bile. This leads to the formation of different corrosion products during stent service, with unknown impacts on the corrosion process. Additionally, stent degradation *in vivo* is a complicated process regulated by several environmental parameters in addition to body fluid composition, including temperature, luminal compression and peristalsis. For instance, in the biliary environment, the stent surface is frequently covered with precipitates and bile duct endothelial cells, which serve as a barrier to further corrosion [39]. Therefore, stent degradation in the human bile duct is not entirely consistent with *in vitro* or animal investigations.

### Antibacterial coatings

Stent restenosis, which severely affects stent support function and longevity, is usually addressed through high-risk percutaneous transhepatic puncture. Restenosis is caused by three key factors: biofilm formation, bacterial adhesion and bile sludge buildup [40]. Bacterial adhesion is frequently present in biliary tract injury [41] and plays a significant role in the development of biofilms, which can result in the buildup of biliary sludge [42].

Following stent implantation, proteins and other chemicals are first adsorbed on its surfaces, followed by bacterial colonization, bile sludge formation and restenosis [43]. The antibacterial stent is implanted when the bacteria did not form a biofilm. This is the perfect time to exert antibacterial effects. Because live bacteria in biofilms are typically surrounded by the extracellular matrix and dead bacteria, which impede antimicrobial drugs from fully penetrating the biofilm. Theoretically, long-term stent opening and successful patency might be achieved by creating biliary stents coated with an antimicrobial coating to inhibit bacterial growth.

#### Silver nanoparticles in antibacterial coatings

With their antibacterial, anti-inflammatory, antiviral and antioxidant capabilities, silver nanoparticles (AgNPs) can cling to and penetrate bacterial cell walls, increasing permeability and causing bacterial cell membranes to disintegrate. AgNPs have hundreds of times the bactericidal effect of regular silver due to their extreme surface effect, quantum size effect and quantum tunneling effect [44, 45]. It is currently the most widely commercialized nanomaterial.

**Figure 3.**
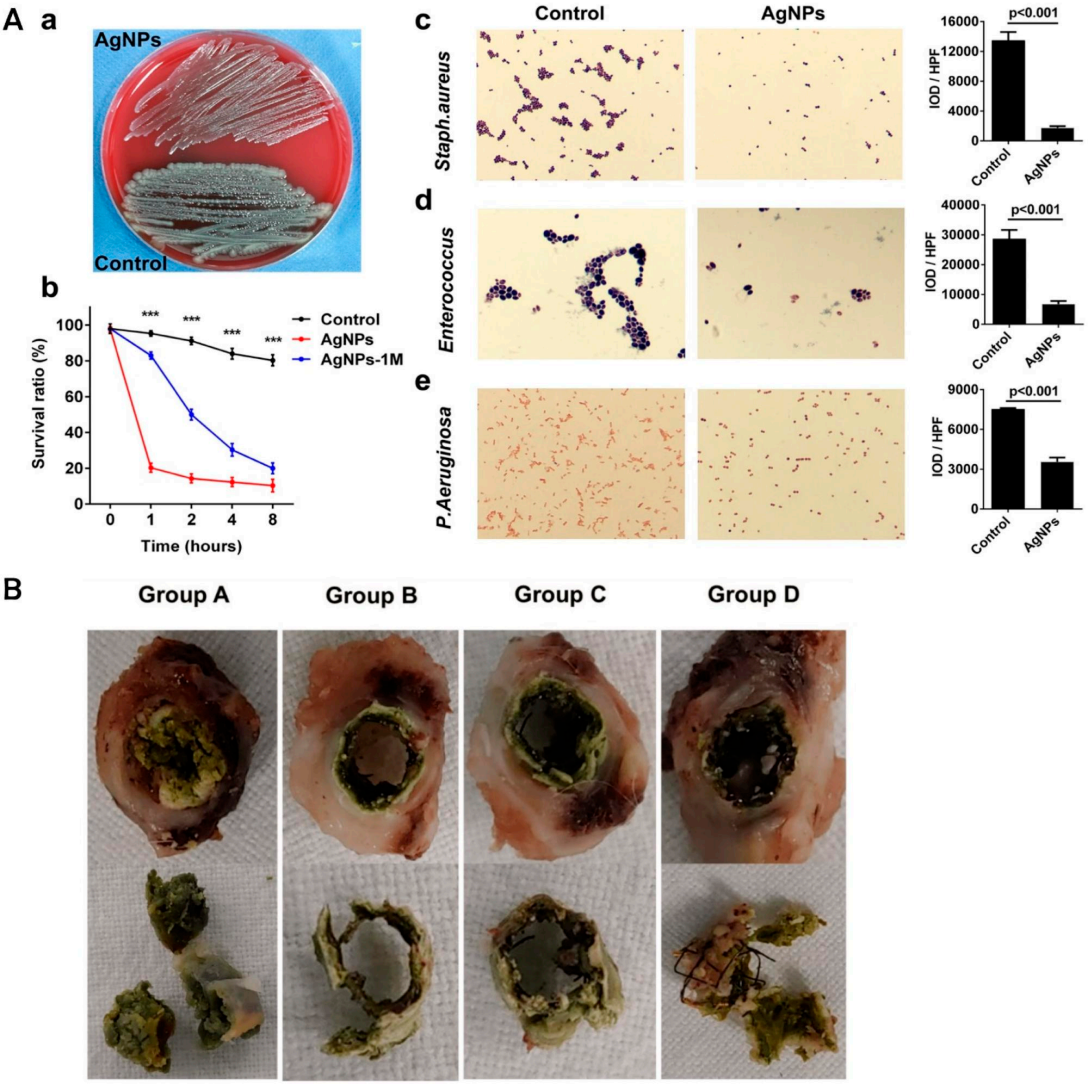
(**A**) Antibacterial function of AgNPs biliary stent *in vitro*. (a) Comparison of number of colonies after 1 h of co-culturing with *E.coli* for AgNPs and controls, respectively. (b) Survival ratios of *E.coli* compared at different time points of AgNPs and *E.coli* co-culturing vs. control (AgNPs-1M: AgNPs biliary stent storage for 1 month). (c) Number of colonies of *S.aureus* compared in AgNPs and *E.coli* co-culturing vs. control (×400). (d) Number of colonies of *Enterococcus* compared in AgNPs and *E.coli* co-culturing vs. control (×400). (e) Number of colonies of *P.aeruginosa* compared in AgNPs and *E.coli* co-culturing vs. control (×400). IOD: integral optical density; HPF: high-power field. Reproduced with permission from Yang *et al.* [46]. (**B**) Degree of biliary sludge formation in different groups. Reproduced with permission from Park *et al.* [47].

Yang *et al.* [46] developed a novel biliary stent coated with AgNPs. An *in vitro* antibacterial assay and implantation of a porcine bile duct stent coated with AgNPs were performed to assess the antibacterial effect of the stent and the prevalence of biliary blockage; furthermore, Ag^+^ release of the Ag-loaded biliary stent placed in phosphate-buffered saline (PBS) was continually monitored. After co-culture of AgNPs with bacteria, the Ag-loaded biliary stents significantly reduced *Escherichia coli*, *Staphylococcus aureus*, *Enterococcus* and *Pseudomonas aeruginosa* when compared to the Teflon biliary stents used as controls (Figure 3A). The control group also consistently had higher positive bile bacterial cultures from 4 to 12 weeks and 24–48 weeks after surgery, of 0%, 33.33%, 50% and 100%, respectively, whereas the AgNPs-coated stents consistently had negative bacterial cultures from 4 to 12 weeks. Finally, the mean time to biliary obstruction (72.37 ± 4.23 weeks) and mean survival time (73.88 ± 4.06 weeks) were considerably longer in the AgNPs-coated biliary stent group compared to the control group (40.13 ± 3.30 weeks and 41.38 ± 3.24 weeks, respectively). The concentration of released Ag^+^ measured using inductively coupled plasma mass spectrometry was at its maximum on the first day (8.52 ± 0.81 μg/l), followed by a significant decrease during the first 10 days (2.86 ± 0.58 μg/l) and gradual decreases after 20, 30, 45 and 60 days to 2.53 ± 0.46, 2.39 ± 0.29, 2.07 ± 0.23 and 1.81 ± 0.17 μg/l, respectively. In addition, results of animal studies revealed that plasma total bilirubin and direct bilirubin levels were significantly lower in the AgNPs biliary stent group than in the control group from 12 to 24 weeks and then to 48 weeks after stent implantation. Thus, Ag-loaded biliary plastic stents exhibited sustained and effective antibacterial activity both *in vitro* and *in vivo*. Ag^+^ release within the AgNPs biliary stent is a powerful and slow process, and the AgNPs biliary stent has a powerful broad-spectrum antibacterial function that can significantly reduce bile duct bacterial infections and effectively maintain the patency of the biliary system *in vivo*.

Park *et al.* [47] loaded polydopamine onto nitinol self-expandable metallic stent, and then immersed it in different concentrations of AgNO_3_ solution (3, 6 and 12 mg/ml), SEMSs with various concentrations of AgNPs coatings (designated groups B, C and D and the blank control as group A) were obtained. The patency of the stent and the formation of biliary mud were examined after placing the four stents in the common bile ducts of rabbits. Hematoxylin and eosin (H&E)-stained sections were used to assess the degree of submucosal inflammatory cell infiltration, the thickness of submucosal fibrosis and the granulation tissue-related percentage of the bile duct cross-sectional area of stenosis. Masson-stained sections were used to look at the degree of collagen deposition. As Figure 3B shows that biliary sludge formation was more prominent in group A than in groups B, C and D. In addition, the mean percentages of granulation tissue area in the four groups A–D were 56.92% ± 9.80%, 21.64% ± 5.61%, 22.69% ± 6.85% and 26.01% ± 7.06%, respectively; the mean thickness of submucosal fibrosis was 0.79 ± 0.20, 0.29 ± 0.10, 0.31 ± 0.09 and 0.39 ± 0.13 mm, respectively; the degree of inflammatory cell infiltration was 3.35 ± 0.75, 2.45 ± 0.68, 2.70 ± 0.66 and 2.85 ± 0.74, respectively; and the degree of collagen deposition was 3.45 ± 0.69, 2.75 ± 0.72, 2.85 ± 0.67 and 2.75 ± 0.64, respectively. The biliary metal stents coated with AgNPs showed a lower degree of submucosal inflammatory cell infiltration, thickness of submucosal fibrosis, granulation tissue-related percentage of bile duct cross-sectional area of stenosis, and degree of collagen deposition compared to the control bare stents. Thus, the AgNP-coated metal self-expanding biliary stent significantly inhibited the formation of extrahepatic bile duct tissue proliferation and cholestasis in rabbits.

However, the effect of AgNPs on biliary stents is not confined to the suppression of bacteria. Previous studies confirmed that the cytotoxicity of AgNPs is dependent on concentration [48], Ag^+^ release regulated by oxygen interactions, particle size and shape and coating [49], in practical applications. Therefore, their cytotoxicity must be given due consideration and the evaluation of AgNPs cytotoxicity in relation to different designs is necessary. For example, strategies, such as avoiding the aggregation of AgNPs [50] and setting slow-release interlayer [51], can reduce the toxicity of AgNPs. In addition, studies on the long-term toxicity of AgNPs have not been reported and their dose-ranging effects are still unclear; thus, additional research is still required to optimize the application of AgNPs in coatings.

#### Chitosan in antibacterial coatings

A range of implanted medical devices, including biliary stents, can incorporate chitosan, a natural polycationic polymer with good broad-spectrum antibacterial characteristics that inhibit biofilm development [52]. Chitosan blocks the growth of microbial cells in two ways: first, by interacting with cell surface anions to create a protective coating that prevents the movement of vital molecules; and second, by blocking RNA and protein synthesis from diffusing into the cell nucleus [53].

Lin *et al.* [54] applied chitosan to the surface of polyethylene biliary stents via methanol precipitation and evaluated the antimicrobial activity of drug-loaded stents in closed PBS flow systems at pH > 7 and in a bile perfusion system with *E.coli* as a model for infected bile. However, the result shown that loaded chitosan stent was ineffective at preventing the *E.coli*.

Liu *et al.* [55] electrophoretically deposited chitosan films on 316 l stainless steel stent substrates, cross-linked the film in a solution of caustic soda and alcohol to reduce swelling of the films. Bacterial adhesion was tested in fresh human bile at 37°C. The result shown that the amount of adherence of enterococci on the surface of the material loaded with uncross-linked chitosan was significantly reduced compared to that of the material not loaded with the coating and no biofilm was seen on the surface of the cross-linked chitosan material. The chitosan coating showed significant antimicrobial activity.

The pH of human bile and different coating material treatment methods may explain the different outcomes of the two studies mentioned above. Chitosan cannot produce efficient antimicrobial activity in an alkaline environment and its antimicrobial effect is constrained by its insolubility in aqueous media. Accordingly, direct application of a chitosan coating to a human biliary environment at a pH greater than seven under normal circumstances will not produce efficient antibacterial effects. Chitosan modification reduces this limitation of its antibacterial properties. By adding quaternary amine groups to chitosan, researchers were able to increase the material’s solubility in aqueous media and induce a permanent positive charge, both of which improved its antimicrobial properties [56]. Additionally, the preparation of chitosan derivatives by swapping amino groups for quaternary amine groups increased its antimicrobial efficiency under alkaline conditions [57]. It is also worth considering how the deacetylation level and molecular weight affect the cytotoxicity and antibacterial activity of chitosan.

#### Antibiotics in stent coatings

Studies have explored physical grafting of antibiotics onto stent coatings. Cefotaxime is a third-generation cephalosporin with antibacterial activity against Gram-positive and Gram-negative bacteria, and it is effective in preventing biliary tract infections after ERCP [58, 59]. Thus, cefotaxime-coated biliary stents could theoretically prevent bacterial attachment and growth, thereby reducing biofilm accumulation and sludge formation. To assess the degree of biofilm development and formation, Gwon *et al.* [60] created cefotaxime-eluting stents and inserted 0% (wt./vol.), 10% (wt./vol.) and 20% (wt./vol.) concentrations of the drug-loaded stents into the bile ducts of dogs. The stents were then submerged in PBS solution to continuously measure the release of cefotaxime. Despite achieving a high percentage of continuous release of cefotaxime in all drug-loaded stents within 1 week and detection of the drug even after 4 weeks, the appearance of structurally similar biofilms on the surface of both drug-loaded and bare stents by SEM. It implied that cefotaxime could not prevent biofilm formation in the canine biliary tract model.

Ampicillin/sulbactam (a combination of ampicillin and beta-lactamase inhibitors) and levofloxacin (a quinolone) have been widely used in clinical practice and have good antibacterial activity. Weickert *et al.* [61] used SEM to study bacterial attachment after coating the surface of polyethylene biliary stents with H*protein A and H*protein A/antibiotics. In SEM, neither the H*protein A and levofloxacin-coated stent nor the H*protein A and ampicillin/sulbactam-coated stent demonstrated significant decreases in adherent material compared to the only coated H*protein A stent used as the control. This indicates that neither antibiotic significantly increases stent occlusion or decreases biofilm formation.

Although some studies have shown that using antibiotics can improve biliary patency, in most studies these antibiotics were administered orally or intravenously rather than directly through the biliary stent. The structural stability of antibiotics in the complex biliary environment and the effective concentration after detachment from the stent may account for the lack of efficacy of local antibiotics. Multiple organisms are typically involved in bacterial infections, and mixed microbial populations, such as aerobic and anaerobic bacteria and fungi, may colonize biofilms [62]. Although the antibiotics chosen in the aforementioned study covered the main bacterial species, it is still possible that certain drug-resistant germs could exist in the biliary environment. Additionally, the timing of drug stent implantation affects outcomes since, once bacteria biofilm formation occurs, antibiotics are ineffective in clearing the biofilm and preventing occlusion [63]. Thus, enhancing the coating preparation process, such as by adding a septum with a unidirectional channel between the drug and the bile to prevent damage to the drug-carrying coating due to contact with the continuous bile flow, may help the coating function more efficiently. Furthermore, the combination of antibiotic function and other antibacterial substances may be an effective way to improve antibacterial efficiency.

### Antitumor coatings

Implanting a biliary stent increases the risk of stent failure due to tissue growth or inward tumor growth through the stent mesh, making the stent difficult to remove or repair. Chemotherapeutic drugs may be applied locally to increase drug concentrations in the tumor environment while reducing systemic exposure and toxicity to organs other than the target [64]. The openness of the stent can be increased by creating drug-eluting stents to be used as drug-delivery systems for local therapy to target drug delivery and prevent tumor growth.

#### Paclitaxel-eluting stents

Paclitaxel (PTX) is one of the most active compounds available for the treatment of human malignancies [65]. PTX stabilizes microtubules and prevents cell growth during mitosis, thus treating cancer [66, 67]. Because of its pharmacokinetic characteristics and steep dose–response to therapeutic effects and toxicity, it is better suited for local administration than systemic administration [68]. PTX-eluting stents may produce high local drug concentrations that are damaging to cancer cells, effectively halting tumor development and spread and improving stent patency.

**Figure 4.**
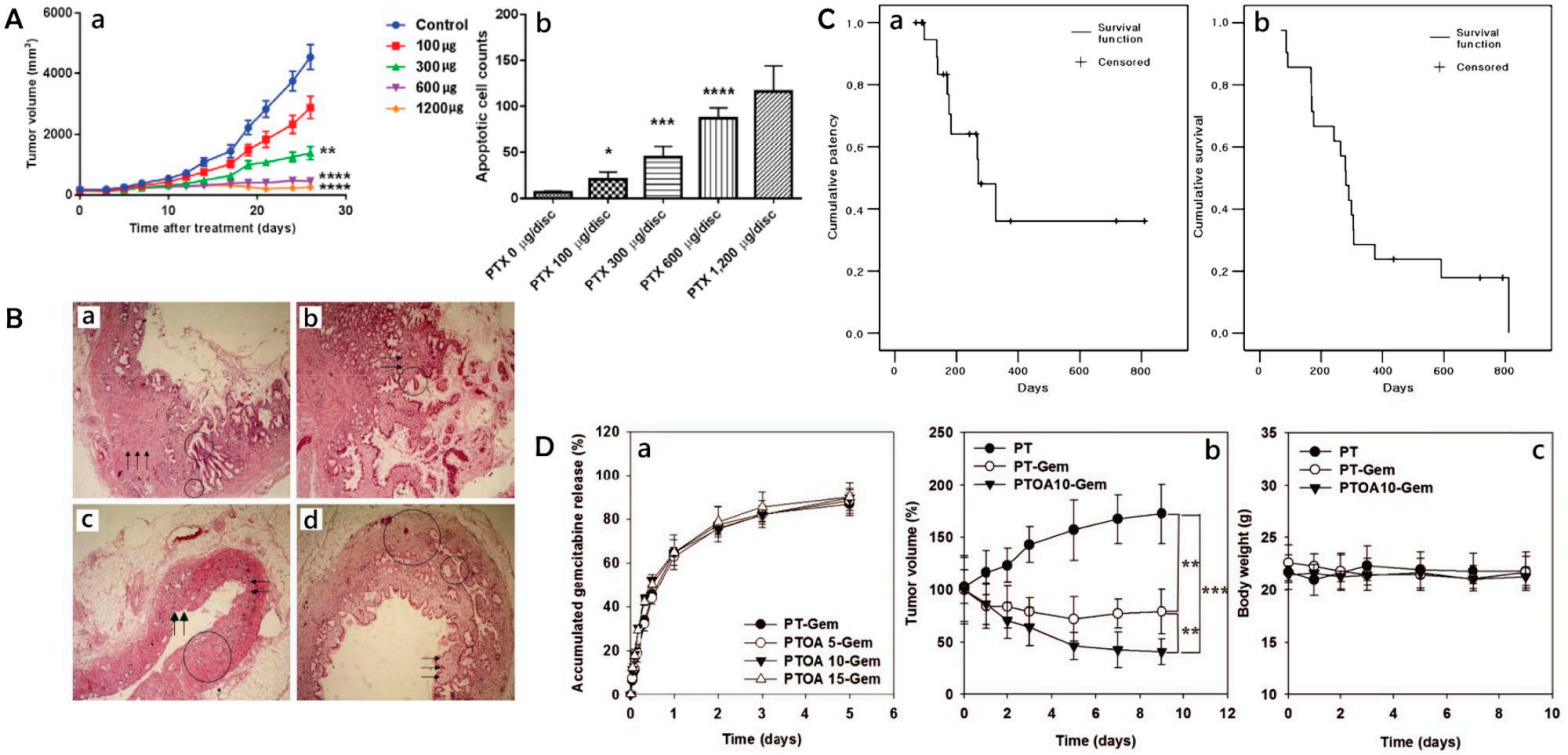
(**A**) (a) Changes in tumor volume as a function of time with various amounts of PTX per disk of membrane; and (b) apoptotic cell count per HPF (×400) with TUNEL staining of harvested tumor tissues. Reproduced from Park *et al*. [69] with permission from Copyright 2018 Spandidos Publications. (**B**) (a) Photomicrograph of bile duct from pig in which a covered metallic stent with 10% wt/v PTX was implanted. Bile duct epithelium is denuded and there is severe fibrosis with wall thickening (arrows), hyperplasia (large circle) and mucinous metaplasia (small circle) (H&E, orig. mag. ×40). (b) Photomicrograph of bile duct from pig in which a covered metallic stent with 20% wt/v PTX was implanted. There is inflammation (arrows), wall thickening and severe mucinous metaplasia (circle) (H&E, orig. mag. ×40). (c) Photomicrograph of bile duct in close proximity to stent with 10% wt/v PTX. Epithelium is denuded (arrowheads) and there are mild mucosal inflammation (arrows) and fibrosis (circle) (H&E, orig. mag. ×40). (d) Photomicrograph of bile duct in close proximity to stent with 20% wt/v PTX. There is moderate mucosal inflammation (arrows), mucinous metaplasia (small circle) and severe wall thickening (large circle) (H&E, orig. mag. ×40). Reproduced from Lee *et al*. [71] with permission from Copyright 2005 the American Society for Gastrointestinal Endoscopy. (**C**) Kaplan–Meier graph showing: (a) cumulative patency of metallic stent covered with PTX-incorporated membrane; and (b) cumulative survival of patients. Reproduced from Suk *et al*. [72] with permission from Copyright 2007 the American Society for Gastrointestinal Endoscopy. (**D**) (a) Release behaviors of GEM from PT-GEM; after membrane insertion, changes in (b) tumor volume and (c) body weight compared at 9 days (*n *=* *3). Reproduced from Seo *et al*. [86] with permission from Copyright 2016 the Polymer Society of Korea and Springer.

Park *et al.* [69] prepared different concentrations of PTX-eluting polyurethane (PU) membranes as stent coatings for a biliary metal stent and assessed their antitumor effect in the mouse model. As shown in Figure 4Aa, on Day 26 after membrane treatment tumor volumes in the 0, 100, 300, 600 and 1200 µg groups were 4536 ± 1307, 2877 ± 1157, 1372 ± 703, 449 ± 349 and 252 ± 325 mm^3^, respectively. No significant difference in body weight of laboratory mice was observed in the groups. As shown in Figure 4Ab, the apoptotic cell count of Terminal deoxynucleotidyl-transferase-mediated dUTP nick end labeling (TUNEL)-stained tumor tissues in the 0, 100, 300, 600 and 1200 µg groups was 6.0 ± 5.0, 20.0 ± 26.0, 45.0 ± 38.0, 87.0 ± 34.0 and 116.0 ± 74.0, respectively (*P* < 0.001). The numbers of tumor cells that underwent apoptosis in the experimental groups were significantly higher than in the blank control group. Therefore, the anticancer effect of PTX-eluting membranes is notable and dose–dependent, but does not exhibit local or systemic toxicity.

PTX has exceedingly poor solubility in aqueous solutions and is highly hydrophobic. Poorly water-soluble drugs like PTX embed themselves in lipophilic tissues *in vivo*, decreasing their bioavailability and increasing bioaccumulation [70]. Thus, increasing the water solubility of these drugs may lead to an increase in drug absorption and a decrease in drug toxicity.

Lee *et al.* [71] investigated the safety of PTX-coated metal stents in animals. PU was mixed with three different PTX concentrations (0, 10 and 20% wt/v) and built on the surface of Ni–Ti biliary stents, then these were placed into the bile ducts of pigs. Histological examination after 4 weeks revealed changes (inflammatory cell infiltration and fibrous response) in the porcine biliary epithelium within acceptable limits with regard to safety. Epithelial detachment, excessive mucin secretion and epithelial hyperplasia were found in samples treated with 10% and 20% wt/v PTX, but transmural necrosis and perforation were not seen in any of the animals (as shown in Figure 4B). The 6-week release of PTX from the stents in PBS was also measured continuously and the *in vitro* release of different concentrations of PTX was similar at 1 and 6 weeks, the stent with 10% (wt/v) PTX was superior in terms of histological changes and drug release effect.

The safety of a PTX-loaded biliary stent was initially demonstrated in a porcine animal model; subsequently, this type of stent was studied by Suk *et al.* [72] by implanting it in 21 human patients diagnosed with unresectable malignant biliary obstruction. The findings demonstrated that drug toxicity associated with PTX did not appear, and the stent did not cause early or serious complications *in vivo*. As shown in Figure 4C, with cumulative patency rates of 100%, 71% and 36% at 3-, 6- and 12-months following stent implantation, as well as cumulative survival rates of 90%, 67% and 29%. When compared to data from previously reported studies on biliary metal stents, such as PU-covered metal stents [73] (233 and 206 days), the mean total stent patency was 429 days, and mean patient survival was 350 days. Therefore, the PTX-coated stents were effective and safe. Notably, the authors of this study added pluronic F-127 as a surfactant to the PTX-containing membrane material. PTX can form a micellar core after incorporation of pluronic F-127, increasing its solubility, metabolic stability and circulation time [64]. In general, animal experiments and *in vitro* simulations are unable to fully recreate cancer cell lines, bile composition, biliary flow environment and other factors in humans. Therefore, results from human experiments are more convincing and offer crucial information to support the transition of PTX-loaded drug stents from theoretical studies to clinical application.

The signaling pathways underlying the antiproliferative actions of PTX-eluting membranes in mice were discovered by Bang *et al.* [74]. PTX controlled hypoxia-inducible factor to inhibit the mammalian target of rapamycin protein and promote apoptosis. Additionally, PTX reduced the expression of proteins, such as CD44, SPARC, matrix metalloproteinase 2 and waveform proteins, that are related to tumor–stromal interaction. Thus, topical delivery of PTX by PTX-eluting membranes activated the rapamycin complex 1-induced apoptosis signaling pathway and prevented angiogenesis, in turn preventing tumor growth in naked mice.

Recent studies on the use of PTX drug-coated biliary stents in humans are summarized in Table 3, with generally confirmed safety but less than adequate efficacy. Human bile is a complex fluid system made up of inorganic substances, bile salts, bile acids, lecithin and cholesterol. The PU used as drug carriers in these studies is vulnerable to erosion and damage in the fluid biliary environment, leading to the formation of microcracks and pores that allow inward tumor growth and result in stent occlusion [64, 75]. This likely inhibited the coated drugs from manifesting their full potential.

**Table 3.** Studies of PTX-loaded coating on biliary stents in humans

Year	Coating carrier	Valid samples	Drug concentration (wt/vol) (%)	Type of study	Results	Ref.
2007	PU	21 cases	10	Multicenter pilot study	Stent occlusion occurred in nine patients. Complications included obstructive jaundice in six, cholangitis in three and one patient showed stent migration with cholecystitis. Mean patency was 429 days and mean survival of patients was 350 days.	[72]
2011	PU	24 cases in drug-eluting stent group and 25 cases in control group	20	Prospective comparative pilot study	In drug-eluting stent group, there were five cases of stent occlusion, three cases of transient cholangitis and one case of pancreatitis. In control group, eight cases of stent occlusion and one case of pancreatitis occurred. Stent patency duration and survival time were not significantly different between the two groups (*P *=* *0.307 and 0.596, respectively).	[76]
2013	PU	58 cases in drug-eluting stent group and 42 cases in control group	10	Prospective comparative study	Stent occlusion caused by tumor growth occurred in 12 patients in the drug-eluting stent group and eight patients in the control group. Stent patency duration and survival time were not significantly different between the two groups (*P *=* *0.116 and 0.981, respectively).	[77]
2017	PU	40 cases in drug-eluting stent group and 32 cases in control group	10	Prospective randomized comparative study	In drug-eluting stent group, there were 14 cases of stent occlusion, compared to seven cases in control group. No discernible differences in the two groups’ stent patency and survival times (*P *=* *0.355 and 0.570, respectively).	[78]

#### Gemcitabine-eluting stents

Gemcitabine (GEM) is a novel nucleoside analogue with cell phase specificity that kills mainly S-phase cells and blocks progression of the cell cycle [80]. In comparison to PTX, GEM is more effective at preventing the proliferation of biliary tract tumor cells and is regarded as a major drug in the treatment of advanced biliary tract cancer [81, 82]. GEM requires phosphorylation to its active metabolite, GEM triphosphate. GEM triphosphate competes with deoxycytidine triphosphate for incorporation into DNA, inhibiting DNA synthesis [83]. Nevertheless, the anticancer effects of GEM triphosphate are minimal because it is rapidly removed by cytidine deaminase and primarily replaced by the inactive metabolite 2'2'-difluoro deoxyuridine [84]. Therefore, substantial doses of GEM are frequently needed to produce therapeutic results, with toxic adverse effects. Targeted drug delivery may improve drug utilization and reduce systemic toxicity.

Chung *et al.* [79] evaluated the safety of GEM-eluting stents. PU membranes containing different concentrations (0%, 10%, 15% and 20% [w/v]) of GEM were loaded on the surfaces of metal stents and implanted into the bile ducts of pigs. Aspartate transaminase (AST), alanine transaminase (ALT), total bilirubin and gamma-glutamyl transferase (γ-GTP) liver function parameters were measured and tissue sections were stained and observed after 4 weeks to assess the effect of the stents on bile duct proliferation. As shown in Table 4, ALT, total bilirubin and γ-GTP were not significantly elevated in all animals after stent implantation. AST was elevated in most experimental animals, but not in a GEM dose–dependent manner, meaning that it was not related to the direct effects of GEM. No significant mucosal hyperplasia, necrosis or perforation was observed in any of the experimental GEM stent groups, but there was significant inflammatory cell infiltration to varying degrees; antiproliferating cell nuclear antigen antibody immunostaining showed that the higher the concentration of GEM, the more pronounced the inflammatory response. Wheatley trichrome staining showed a similar degree of submucosal fiber response in all porcine stent segments, suggesting that the fiber response was not dependent on GEM concentration. After 2 weeks of incubation in PBS with the drug-coated membranes, the average release of 10%, 15% and 20% GEM membranes was 149 mg (64.8%), 223 mg (68.2%) and 318 mg (74.3%), respectively. After 4 weeks, the average medication release was 156 mg (67.8%), 235 mg (71.9%) and 337 mg (78.7%), respectively. Drug release did not significantly increase throughout this time. These findings demonstrated that GEM-eluting stents exhibited no significant stent-related complications in porcine bile ducts and could be used safely in human bile ducts. Furthermore, 10% GEM, which may be the concentration most suitable for clinical application, produced only minor histological changes in the stented segments and adjacent tissues. Notably, the study also established silver hydroxyapatite membranes and silicone membranes with PU on the inside and outside of the drug-carrying coating, respectively. The silica membrane containing silver hydroxyapatite was designed to prevent the occurrence of inflammation. The PU membrane was designed to prevent GEM from entering the stent lumen, reduce fluctuations in drug release during bile flow and reduce damage to the stent surface by bile.

**Table 4.** Laboratory test results after stent placement^a^^,^^b^

	AST (IU/l)			ALT (IU/l)			Bilirubin (mg/dl)			γ-GTP (IU/l)		
	Wk 0	Wk 2	Wk 4	Wk 0	Wk 2	Wk 4	Wk 0	Wk 2	Wk 4	Wk 0	Wk 2	Wk 4
0% GEM-1	60	NA	106	40	NA	86	0	0	0	142	NA	112
0% GEM-2	44	35	430	32	29	65	0	0	0	13	6	41
10% GEM-1	81	90	46	61	32	45	0	0	0	57	48	32
10% GEM-2	49	57	95	56	37	60	0	0.01	0	58	71	76
15% GEM-1	35	41	52	52	35	36	0	0	0	51	63	76
15% GEM-2	35	84	60	33	31	53	0	0	0.01	48	62	38
20% GEM-1	49	48	110	35	32	30	0.01	0	0	21	26	29
20% GEM-2	46	67	57	45	27	44	0	0.01	0	19	75	65

a ALT, alanine transaminase (40–106 IU/l); AST, aspartate transaminase (normal range, 13–47 IU/l); bilirubin (normal range, 0.0–0.2 mg/dl); γ-GTP, gamma-glutamyltransferase (normal range, 25–78 IU/l); GEM, gemcitabine; NA, not available; Wk, week.

b Reproduced from Chung *et al*. [79] with permission from Copyright 2011 Journal of Gastroenterology and Hepatology Foundation and Blackwell Publishing Asia Pty Ltd.

GEM has strong hydrophilicity, hindering its crossing of cell membranes and allowing diffusion out of liposomal bilayers. As a result, it often has a low drug encapsulation efficiency and undesirably fast drug release [85], thereby restricting its use in drug-delivery systems.

Seo *et al.* [86] combined GEM with oleic acid (OA) and ethane (PT) to create binding membranes, where OA served as a tissue-penetration enhancer to improve GEM admixture to cancer cells and PT served to lessen rapid initial rupture and washout of water-soluble GEM. To evaluate the release behavior of the drug-loaded film in the aqueous phase with various levels of OA content, the binding film was deposited on the surface of the metal biliary stent and placed in PBS. As shown in Figure 4Da, regardless of the amount of OA in each membrane, ∼50% of the burst release was observed in all membranes after 12 h, and at 5 days ∼85% of the GEM had been released from all membranes. To study the *in vivo* inhibitory effect of subcutaneous cholangiocarcinoma growth using a mouse model, a pure PT film, PT-GEM film and PT-GEM film with OA were surgically placed into the lower part of the tumors of nude mice. Tumor size and volume changes were continually observed after the surgery. As can be seen from Figure 4Db, the tumor volume of the PT film group continued to increase by roughly 175% after 9 days, whereas tumor size was significantly reduced by PT-GEM and PT-GEM film with OA after just 1 day. Considerable decreases in tumor volume within 9 days were of up to 20% (PT-GEM) and 45% (PT-GEM with OA) due to both the significant inhibitory impact of GEM and the reinforcement of this effect by OA as a penetration enhancer, it was shown in Figure 4Dc. Further evidence of the safety and non-toxicity of GEM membranes was provided by the fact that none of the three types of membranes showed *in vivo* toxicity during the test period and no mice experienced weight loss or died.

The signal transduction mechanisms of the antiangiogenic and antiproliferative properties of GEM-eluting membranes were discovered in nude mice with bile duct cancer [87]. GEM enhanced the protein levels of c-CBL E3 ubiquitin ligase and triggered the ubiquitination and degradation of the epidermal growth factor receptor (EGFR), thereby reducing the downstream functions of EGFR, including tumor cell proliferation, angiogenesis and metastasis.

Nevertheless, safety concerns remain around antitumor-eluting stents due to reports of mucin hypersecretion and bile duct epithelial separation, as well as the detrimental effects of anticancer drug-eluting coatings on normal cells. Therefore, it is crucial to weigh the advantages and disadvantages of local antitumor effects while choosing medications and designing doses so as to strike a balance between safety and efficacy.

Table 5 displays research findings on various biliary stents that were implanted with antitumor medications in animal models. It is evident from study cases spanning more than 10 years that several medications have demonstrated efficacy in animal models and some have also demonstrated good safety. The effectiveness and safety of drug coatings in animal models are crucial, serving as both valuable benchmarks and essential conditions for upcoming human trials. The vast majority of biliary stents used today are constructed of plastics or metals that their own do not slow tumor growth does not slow tumor growth. Due to their excellent mechanical characteristics and biocompatibility, biodegradable Mg and its alloys are frequently employed in medical procedures. A recent study [88] showed that Mg had good inhibitory effects on both human hepatic bile duct cancer cells and mouse hepatocellular carcinoma tumors (H22 tumors). Thus, using biodegradable Mg as a stent substrate in conjunction with an antitumor coating is anticipated to further enhance the tumor-inhibiting effect of biliary stents.

**Table 5.** Studies of antitumor drug-coated stents in animal models

Year	Coated drug	Coating carrier	Cells	Experimental animals	Security	Validity	Ref.
2011	GEM	PU	N/A	Pig	No significant stent-related complications were observed and stents containing 10% (w/v) GEM resulted in milder degree of inflammation, it may be appropriate for clinical application.	N/A	[79]
2013	PTX	Poly-L-lactide acid	N/A	Dog	There was no significant difference in liver function between the drug-loading group and the control group, and no bile leakage occurred.	PTX significantly inhibited activation and proliferation of myofibroblast and excessive collagen deposition in biliary-enteric anastomosis, and prevented overhealing and scar contracture of anastomotic stoma.	[89]
2013	Sorafenib	Poly(ε-caprolactone)	HuCC-T1	Mice	N/A	Sorafenib-loaded PCL films are effective in inhibiting angiogenesis, proliferation and invasion of cancer cells.	[90]
2017	GEM	PU	HuCC-T1	Mice	Drug-eluting film did not show any *in vivo* toxicity to mice and no mice showed weight loss or died.	Drug-eluting membranes significantly inhibited tumor growth in tumor-bearing mice.	[86]
2017	Vorinostat	Poly(DL-lactide-co-glycolide)	HuCC-T1	Mice	N/A	Vorinostat-eluting nanofiber membranes showed significant antitumor activity against cholangiocarcinoma cells *in vitro* and *in vivo*.	[91]
2018	PTX	PU	CT26	Mice	No local or systemic toxicity observed in membranes of all levels of PTX.	PTX membranes showed significant dose–dependent antitumor effects.	[69]

### Stone-dissolving coatings

Bile duct stones are a common condition that can cause cholangitis or acute pancreatitis. Primary bile duct stones are mainly composed of bilirubin, while secondary bile duct stones are mainly composed of cholesterol [92]. Sodium cholate (SC) and ethylenediaminetetraacetic acid (EDTA) can dissolve cholesterol and pigment stones, respectively. Furthermore, the use of SC and EDTA leads to an excellent prognosis and low incidence of major side effects. Endoscopic retrograde cholangiography combined with endoscopic sphincterotomy is an established treatment for common bile duct stones [93, 94]. Although most patients with common bile duct stones can be successfully treated with these conventional methods, endoscopic biliary stenting is a more suitable option for elderly patients and patients with severe comorbidities [95], where other endoscopic or surgical procedures may pose an unacceptably high risk for the treatment of common bile duct stones. In such cases, it is anticipated that combining medications with biliary stents will stimulate gallstone breakdown and increase therapeutic effectiveness.

**Figure 5.**
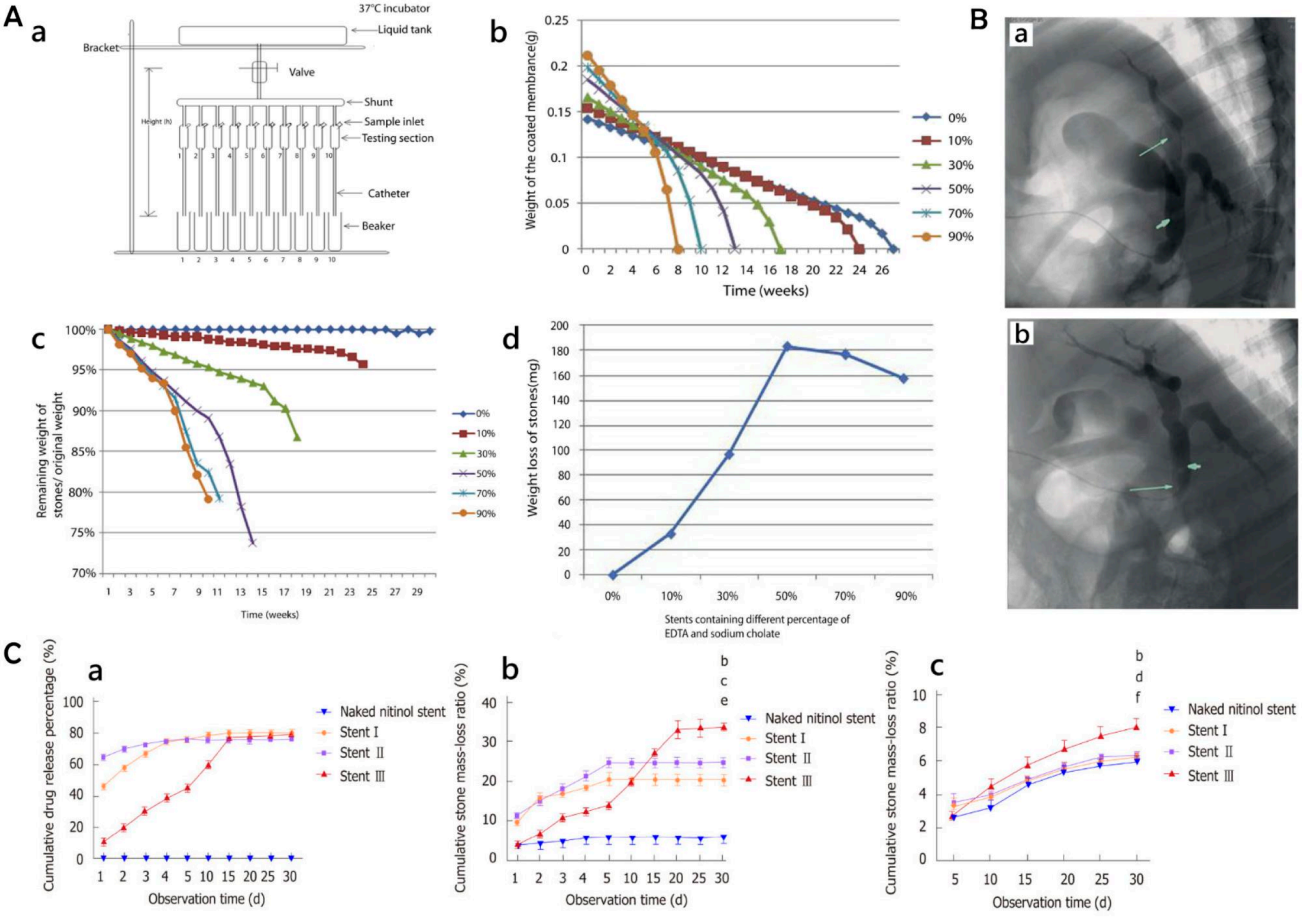
(**A**) (a) Novel *ex vivo* bile duct model; (b) degradation curve of stents containing different percentages of EDTA and SC in *ex vivo* porcine perfusion model; (c) curves of percentage of remaining stones weight over time increased; and (d) total weight of stones dissolved by different stents. Reproduced from Cai *et al*. [96] with permission from Copyright 2014 the American Society for Gastrointestinal Endoscopy. (**B**) Cholangiograms of a pig into which a human common bile duct stone (arrowhead) and stent (arrow) coated with a membrane containing 50% EDTA and SC were inserted: (a) and (b) are 4 and 16 weeks after stent placement, respectively. Reproduced from Cai *et al*. [97] with permission from Copyright 2015 Rights Managed by Georg Thieme Verlag KG Stuttgart. (**C**) Drug release behavior and stone-dissolving efficacy of different stents: (a) drug release curves of three types of drug-eluting stents and stents without drug load; (b) stone mass loss curves of three types of drug-eluting stents and stents without drug load in still buffer; and (c) stone mass loss curves of three types of drug-eluting stents and stents without drug load in flowing bile. Naked fully covered SEMS vs. Stent III, ^a^*P*<0.05, ^b^*P*<0.01; Stent I vs. Stent III, ^c^*P*<0.05, ^d^*P*<0.01; Stent II vs. Stent III, ^e^*P*<0.05, ^f^*P*<0.01. Reproduced from Huang *et al*. [98] with permission from Copyright 2019 Baishideng Publishing Group Inc.

A novel drug-eluting stent was created by Cai *et al.* [96] in which different concentrations (0%, 10%, 30%, 50%, 70% and 90%) of SC and EDTA (1:1 molar ratio) were loaded onto the surfaces of the plastics to test stent efficacy in dissolving bile duct stones through a new *ex vivo* bile perfusion model, as shown in Figure 5Aa.

All stents containing EDTA and SC have the ability to dissolve gallstones. However, the higher the drug content, the better the stone dissolution efficiency and the faster the stent degradation rate. Stents without drugs have no stone-dissolving ability, but showed the slowest degradation, with a total degradation time of 31 ± 1.7 weeks, as shown in Figure 5Ab and c. Greater total stone weight reduction (183 ± 49, 33 ± 17 and 97 ± 31 mg, respectively) and percentage (26.2% ± 4.4%, 4.3% ± 2.1% and 13.2% ± 5.1%, respectively) were achieved at 13.0 ± 1.2 weeks of complete degradation for the 50% EDTA and SC stents compared to the 10% and 30% EDTA and SC stents. Although the 70% and 90% EDTA and SC stents showed better lithotripsy rates before complete biodegradation of the envelope, total stones and percentage weight loss were slightly lower in these two groups than in the 50% EDTA and SC stents due to faster degradation (177 ± 36 , 158 ± 32 and 183 ± 49 mg total stones, respectively, and 26.2% ± 4.4% vs. 20.8% ± 7.6% and 20.9% ± 9.1%, respectively). Overall, among all stent groups the 50% EDTA and SC stents had the best stone weight reduction, as shown in Figure 5Ad.

The safety and effectiveness of these novel stents were then assessed in a pig gallstone model based on *in vitro* investigations [97]. The uncoated stents, 0% EDTA/SC-loaded stents (i.e. PLA- and PCL-loaded matrix only), and 50% EDTA/SC-loaded stents were inserted into experimental pigs along with human gallstones. On the second cholangiogram following stent insertion, bile duct stones in the two pigs with 50%-coated stents had significantly reduced in diameter (>50%), as shown in Figure 5B, compared to relatively small to moderate changes in stone size (30%) in the other two groups of animals. The dilated common bile ducts of all animals had smooth external and interior walls that lacked ulceration and hyperplasia. Aside from slight inflammatory cell infiltration, none of the animals had any symptoms of necrosis, atrophy or alterations in epithelial layer thickness. The weight of all stones decreased 6 months after the stent was inserted. The total weight decrease of stones was greater in the 50%-coated stent group than in the uncoated and 0%-coated groups (269 ± 66, 179 ± 51 and 156 ± 26 mg, respectively), and the percentage change in body weight was also different (44%±15%, 28%±8% and 23%±3%, respectively). Additionally, the stone fragmentation rate in the 50%-coated group was higher than in the uncoated and 0%-coated groups (3 vs. 1 vs. 1, respectively). Therefore, the breakdown of common bile duct stones can be effectively and safely promoted by these novel stents coated with lysis agents.

To achieve more effective drainage and resolve the issue of drug stent coating detachment and drug burst release, Huang *et al.* [98] modified the above stone-displacing stent. SC and EDTA were placed on the stent in a 1:1 molar ratio and PCL was utilized as the drug carrier. Dip-coating (Stent I), coaxial electrostatic spinning (Stent II) and dip-coating combined with electrostatic spinning (Stent III) were used to create three different types of drug-eluting stents. To choose the optimal manufacturing technique, the drug release performance and stone-dissolving efficacy of the stent were first assessed *in vitro*. The selected stone-dissolving stents were then further implanted into the pig common bile duct for biosafety testing. The three different types of drug-eluting stents were immersed in PBS and the release of SC and EDTA from the buffer was continuously measured. Four different types of stents were co-incubated with bile duct stones (all 100 mg mass) in PBS and the mass loss rate of the stents at 1, 2, 3, 4, 5, 10, 15, 20, 25 and 30 days of incubation was used to estimate the stone dissolution effect. Using 3DP technology, a human-scale *in vitro* model of the common bile duct was created to recreate the bile flow environment. Stents and bile duct stones (100 mg mass unit) were inserted into the model and 1000 ml of human bile was perfused each day. After 5, 10, 15, 20, 25 and 30 days of bile perfusion, the mass loss rate of stones was assessed. Serum was taken for testing 15 and 30 days before and after the stents were implanted in male minipigs. After a month of follow-up, they were all euthanized and samples of the gallbladder, common bile duct, intestinal wall, liver and kidney were gathered for staining and examination. All drug-eluting stents had good patency and smooth surfaces. For the same medication-loading volume and drug-loading substance, Stent II exhibited the lowest drug-loading capacity. As can be seen from Figure 5Ca, Stents I and II had considerable burst drug release in the first 5 days, but Stent III achieved controlled and sustained drug release over 30 days. In the static buffer, the final stone loss rates were 5.19%±0.69%, 20.37%±2.13%, 24.57%±1.45% and 33.72%±0.67% for each of the bare stent and Stents I, II and III, respectively, as shown in Figure 5Cb; in flowing bile, the final stone loss rates for each group were 5.87%±0.25%, 6.36%±0.48%, 6.38%±0.37% and 8.15%±0.27%, respectively, as shown in Figure 5Cc. Thus, Stent III had the highest stone loss in both the stationary and flowing environments. In pigs, these findings revealed no necrosis or fibrosis of the gallbladder or common bile duct, and a normal liver, kidney and duodenal wall, with no differences in serological or histological analyses (*P *>* *0.05). Therefore, this new, improved SC and EDTA-eluting biliary stent is effective in reducing bile duct stones *in vitro*.

Thus, human bile and gallstones were used to replicate the human biliary environment and the biliary stent equipped with a stone-dissolving coating produced positive results both *in vitro* and in animal trials, proving its efficacy and viability. It’s worth noting that the dissolution behavior of stones is necessarily of in-depth study, and if there is a large amount of uneven dissolution, then this process may bring safety risks.

### X-ray visibility coating

Biliary stents often need to be implanted into specific sites through endoscopy, and imaging techniques are indispensable. Adequate radiation visibility of stents is of great clinical significance for accurately implantation and localization of biliary stents, and observation of stent status (such as displacement and degradation). Computed Tomography (CT) is the most used modality to assess normal positions and evaluate patients suspected of stent complications [99]. Biliary stents made of traditional radio-opaque metal (such as nitinol or stainless-steel alloys) can be easily observed under CT. The polymer stents suffer from a lack of radiopacity and cannot be visualized.

For this problem, one strategy is to add a radiopaque substance (Like BaSO_4_) as a contrast agent to the polymer [100], which can be directly used as a base material for biliary stent when mixed with the polymer. Another strategy is to indirectly visualize stents by placing radiopaque metal markers on both ends of the stents, such as gold, platinum and tantalum, But these metallic markers only allow visualization of the stent position and not the stent itself, without providing information regarding the extent of the degradation [101]. In addition, coating the surface of the biliary stent is an innovative idea.

Tanimoto *et al.* [102] used the copolymer of L-lactide and ε-caprolactone (70:30) to prepare a biodegradable biliary stent and coated it with a layer of BaSO_4_ to make it can be detected by radiography. Biliary stents coated with barium sulfate were inserted into the common bile ducts of 11 pigs, and CBD and liver were removed for histological examination 6 months after surgery. The results showed that the excisional CBDs had a normal tissue structure, with only minor inflammatory changes, indicating that the radiopaque biodegradable stent had no harmful effects on the bile duct itself.

**Figure 6.**
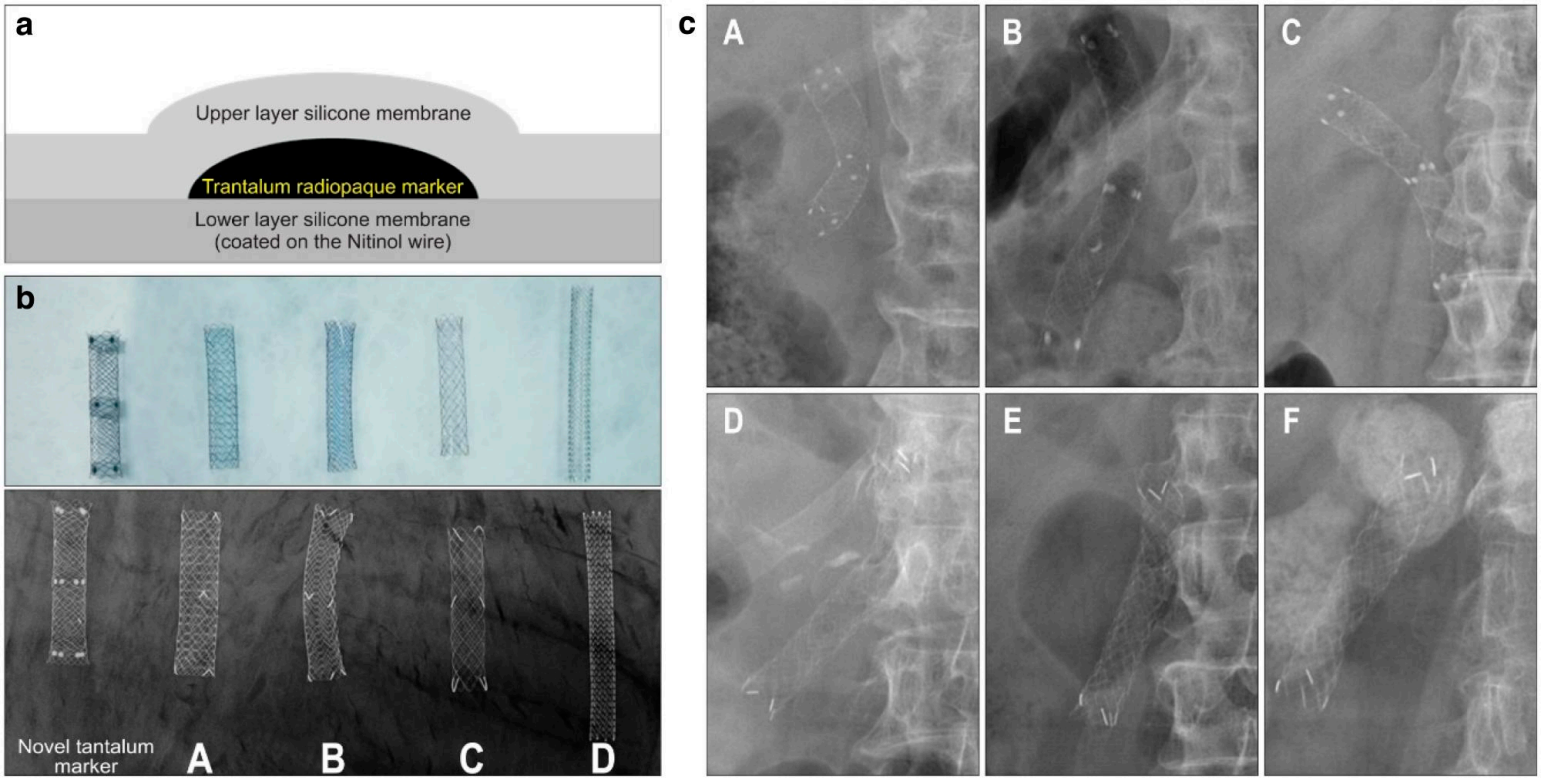
(**a**) Schematic image of a novel tantalum marker; (**b**) radiographic images of nitinol alloy stents with different metal markers. All metal markers were visualized in radiographs (A, NitiS, Taewoong Medical; B, Hanaro, M.I.T. Tech; C, EGIS, S&G Biotech; and D, Zilver, Cook Medical); (**c**) Fluoroscopic images of tantalum and gold markers. Tantalum markers (A–C) were more clearly visible in fluoroscopic images than gold markers (D–F). Reproduced from Park *et al*. [103] with permission from Copyright 2019 the Korean Society of Gastroenterology, the Korean Society of Gastrointestinal Endoscopy, the Korean Society of Neurogastroenterology and Motility, Korean College of Helicobacter and Upper Gastrointestinal Research, Korean Association the Study of Intestinal Diseases, the Korean Association for the Study of the Liver, Korean Pancreatobiliary Association and Korean Society of Gastrointestinal Cancer.

Park *et al.* [103] proposed a strategy to construct tantalum marker coatings on the surface of stents to improve X-ray absorption of SEMSs, and evaluated the safety and visibility of tantalum markers. First, the silicone/xylene mix is sprayed directly onto the surface of the nitinol stent using an ultrasonic spray machine to form a thin coating, then the tantalum metal powder is sprayed using an ultrasonic spray machine to form tantalum dots on the silicone film, and the tantalum dots are covered in the same way with silica gel, as shown in Figure 6a. Finally, the coating with a maximum thickness of 200 µm is obtained, and it contains 12 tantalum markers with a diameter of 2 mm (four at the center, distal and proximal ends of the stent). Five tantalum markers were implanted in the stomachs of three beagle dogs, and abdominal radiographs were taken at 0, 2, 4, 6, 8, 24 and 33 h after all the markers were excreted, and the weight of the excreted markers was compared with that of the original markers. Then, X-ray microscopic images of tantalum markers were compared with those of gold and platinum markers, and the opacity of each marker was measured by ImageJ software. Finally, the radiographic images of six patients who underwent biliary SEMS placement using tantalum marker nitinol SEMSs (*n *=* *3) or gold marker nitinol SEMSs (*n *=* *3) were compared with respect to marker brightness on fluoroscopic images. It was found that none of the collected markers were damaged and the beagle weight did not change (weight recovery vs. original weight: Beagle A, 1495 mg vs. 1492 mg; Beagle B, 1182 mg vs. 1167 mg; Beagle C (1652 and 1627 mg, respectively)); X-ray opacity tests showed that the metal marker was clearer than nitinol; and the tantalum marker had a higher brightness score than the other markers, as shown in Figure 6b. The average brightness and total brightness scores of tantalum markers (226.22 and 757, respectively) were higher than those of gold markers (A. 209 and 355, respectively; B. 204.96 and 394, respectively; C. 194.34 and 281, respectively) and platinum markers (203.6 and 98, respectively). The tantalum marker has a much larger area on the X-ray plate than other markers. On fluoroscopic images, tantalum markers were more clearly visible than gold markers, as shown in Figure 6c. The brightness and total brightness scores of tantalum markers in human bile ducts (41.47 and 497.67, respectively) were higher than those of gold markers (28.37 and 227, respectively). These results imply that the establishment of a coating containing tantalum markers helps to improve the light permeability of the stent, and tantalum markers in the coating are rarely absorbed by the gastrointestinal tract.

The safety of contrast media is the biggest problem faced in the process of use. Adding contrast agents to the inside of the coating is a smart and efficient strategy compared to coating directly on the surface. The contrast agent is not in direct contact with the external environment, thus reducing the damage to the body. The influence of contrast agent itself on the performance of biliary tract and biliary stent cannot be ignored. For example, some scholars have pointed out that there are potential complications from the presence of barium sulfate in the biliary tract, barium suspension may be cause or contribute to stent occlusion [104]. Therefore, when using contrast agent, its dosage, loading method and other factors still need to be cautious, and the problems, such as contrast agent dissolution and leakage that may occur in the biliary tract environment and their consequences should be prospectively considered. Finally, the long-term observation of contrast agent coating in animal experiments will draw more reliable conclusions about its safety, which will become an important safety premise for clinical trials.

### Antistent migration coating

**Figure 7.**
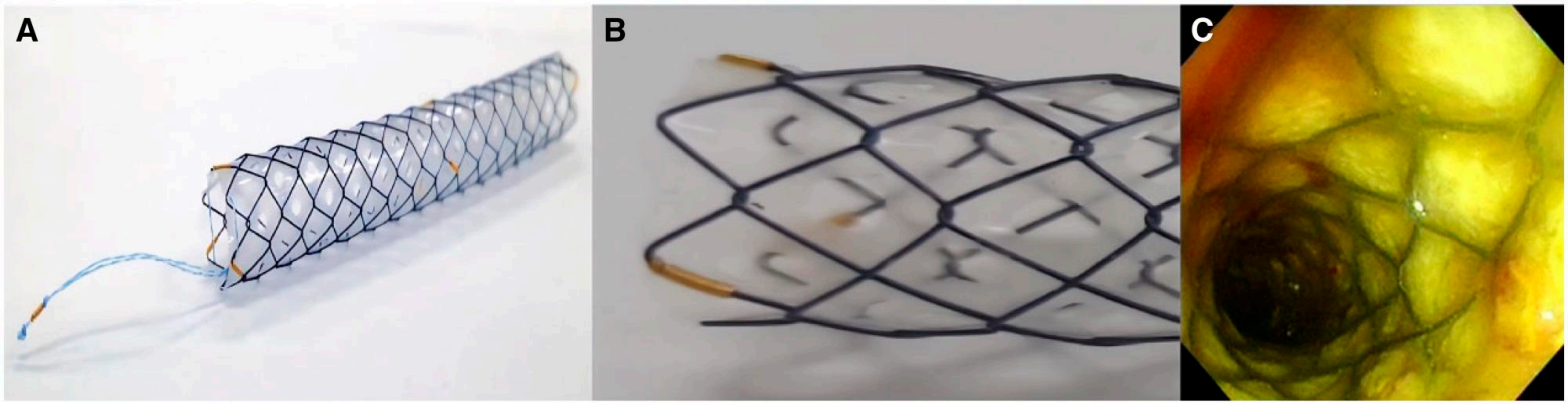
(**A, B**) Biliary stent with antimigration functional coating; (**C**) the biliary stent with antimigration functional coating was fixed to the bile duct wall. Reproduced from Kobayashi [107] with permission from Copyright 2019 Baishideng Publishing Group Inc.

A key problem limiting the safety and effectiveness of long-term stent insertion is stent migration. Stent migration rates of 5%–10% have been reported in numerous investigations, with distal migration seen in 3%–6% of patients [105, 106]. In general, improving the migration of biliary stents focuses mainly on changes to the structural design, such as the development of stents with migration-resistant anchor flaps [10]. Additionally, a recent study has shown that coating technology also appears to be an effective means of addressing stent migration. Kobayashi *et al.* [107] reported an interesting design of covering the surface of the SEMS with a membrane material and drilling small holes in each bracket unit, as shown in Figure 7A and B. When the stent was placed at a joint connected by a side branch, bile poured into the stent through the pores in the membrane. Small holes lower the membrane tension, which fixes the implanted stent to the surrounding tissue and prevents its migration. This new biliary stent was implanted in patients with bile duct obstruction without complications, such as pancreatitis, bleeding or cholangitis and jaundice improved in all patients with malignant strictures, proving their safety. Cholangiography showed that this new biliary stent was well fixed to the bile duct wall, as shown in Figure 7C.

The small holes in the coating not only play an antimigration role, it may also have an inhibitory effect on endoward-growing tumors. This inspired us to change the material of the covering film, the distribution and pore size of the pores on the film or load specific functional active substances onto the film, so that the stent not only has antimigration function but also has more capabilities.

### Functional composite coatings

In the design of multilayer membrane structure, active agents and pro-permeation agents can be added to different membranes to improve the utilization rate of main functional substances, but the overall function of the coating is still single. The role that a single functional coating can play in the real application environment is limited. Therefore, designing a structured composite coating by loading different functional substances into each layer of the film separately may achieve the combination of different functions.

**Figure 8.**
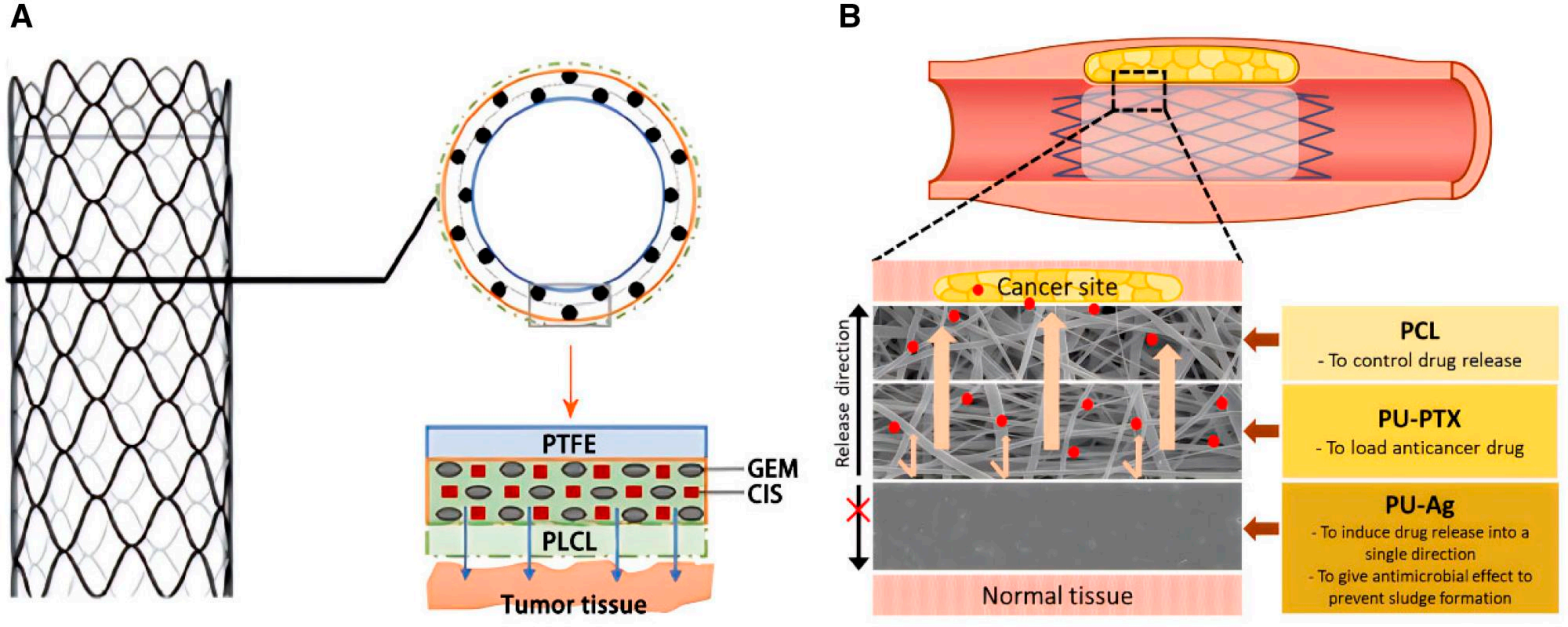
(**A**) Drug-eluting biliary stent loaded with GEM/CIS combination coating. Reproduced from Xiao *et al*. [108] with permission from Copyright 2020 Baishideng Publishing Group Inc. (**B**) Biliary stent with dual anticancer and antibacterial effect composite coating. Reproduced with permission from Rezk *et al*. [109].

Using a hybrid electrospinning technique, Xiao *et al.* [108] loaded GEM and cisplatin (CIS) on four drug-eluting stents at different loading rates (5%, 10%, 15% and 20%), including single-drug stents (poly-L-propylene-caprolactone (PLCL)-GEM and PLCL-CIS)) and two-drug stents (PLCL-GC, GEM: CIS = 1:1). As shown in Figure 8A, the drug-loaded stent consisted of a three-layer, composite functional coating: the inner polytetrafluoroethylene membrane layer ensures the unidirectional release of drugs to the tumor tissue side to avoid fluctuating release and bile flow, leading to drug loss; the middle layer is loaded with two antitumor drugs, GEM and CIS, using PLCL as the carrier; and the outermost layer is a pure PLCL membrane to prevent the burst release of the loaded drugs. Drug release in PBS buffer at specific time intervals from 1 to 30 days was determined using high-performance liquid chromatography. The release of GEM from PLCL-GEM was divided into three phases: the initial rapid release phase (0–10 days, cumulative release of 75% of the total drug loading); the middle stable phase (11–20 days, stable slow release of 85%); and the late plateau phase (21–30 days, near-complete release of ≥95%). CIS release from PLCL-CIS consisted of two phases: the initial phase (Days 0–7, cumulative release of 15%–20%) and the homogeneous stabilization phase (Days 8–30, cumulative release of 25%–35%). The release pattern of both drugs from the two-drug stent (PLCL-GC) was similar to that of the single-drug-loaded nanofibers (PLCL-GEM and PLCL-CIS) albeit with slightly slower release rates. Furthermore, the antitumor effect of a 10% drug-loaded polymeric membrane drug release solution on EGI-1 cells over 30 days was observed. The single-drug-loaded membrane demonstrated inhibition in the early stage and the effect of PLCL-GEM was stronger than that of PLCL-CIS; however, the effect of PLCL-GEM diminished over time starting from Day 10 and significantly diminished in the late stage. The effect of PLCL-CIS was relatively weak but did not have a significant decreasing peak in the middle and late phases but was maintained and gradually decreased to some extent. This is consistent with the release characteristics of GEM and CIS in polymeric membranes, where PLCL-GC polymeric membranes led to good inhibition due to the synergistic effect of the two drugs. PLCL-GC polymeric membranes were stronger than mono-drug polymeric membranes in the early stages, while mono-drug polymeric membranes remained effective in the later stages, and the overall inhibition rate was ∼30%. The drug release medium of PLCL-10% GC inhibited the viability, migration and invasion of EGI-1 cells by GEM and CIS released on nanomembranes during the 30-day drug release period. All of the observed effects were stronger in the first 10 days, then gradually weakened and remained unchanged in the final 10 days. Different types of drug-eluting membranes were surgically implanted beneath tumors in BALB/c nude mice, and body mass and tumor volume were measured every 2-day after implantation. Visually, tumor size in the drug-loaded group was smaller than in the unloaded and control groups. All drug-loaded membranes gradually inhibited tumor growth; PLCL-GC showed the best inhibition effect from the tumor growth curve. Additionally, different types of stents were implanted into the common bile ducts of pigs and serum specimens were collected on Days 0 (preoperative), 1, 3, 7, 14 and 28 to determine serum white blood cell counts and levels of ALT, AST, total bilirubin, γ-GT, amylase and creatinine to evaluate liver and kidney function, and inflammatory response after stent placement. Four weeks after stent placement, tissue specimens of the bile duct, liver, kidney and duodenum were taken for histological examination and general morphological changes of the catheter mucosa and stents were evaluated. All stents were successfully placed into the porcine bile duct without procedure-related complications. Serum ALT, AST, γ-GT and amylase levels were elevated 1 week after stent placement and showed mild to moderate abnormalities. However, these indicators gradually returned to normal after 4 weeks. The general appearance of the bile duct mucosa was similar in all groups and no ulceration, perforation or necrosis were observed. In addition, no significant luminal dilatation, wall thinning or mucosal hyperplasia were observed in the stented bile duct segments. No granulation, atrophy or necrosis were observed in any group and no histological changes were observed in the duodenum, liver or kidney. *In vivo* experiments confirmed the good safety and biocompatibility of the stents in the normal porcine bile duct. Thus, this innovative biliary stent coated with PLCL-GC offers long-lasting local drug release and antitumor activity *in vitro* and in animals, and it is both safe and feasible for application.

Rezk *et al.* [109] designed a three-layer structure of biliary stent coating material, as shown in Figure 8B. The innermost layer of the PU membrane was loaded with different concentrations (1%, 3% and 5%) of AgNPs in direct contact with the luminal fluid, which functions to inhibit the formation of biofilm on the inner surface of the membrane and acts as a physical barrier to direct the release of the drug to the tumor site. An intermediate layer, consisting of PU nanofibers loaded with PTX, exerts antitumor effects. The outermost PCL layer acts as a physical barrier to control the initial burst release of PTX and to improve stent mechanical properties. The zone of inhibition test was used to examine the inhibitory activity of *S.aureus* and *E.coli* against pure PU membranes and PU membranes loaded with various doses of AgNPs. Compared to the control, the inhibition zones of PU membranes loaded with 1%, 3% and 5% AgNPs gradually increased against both bacteria and the survival rate of *E.coli* continued to decline after 24 h of incubation with the AgNPs-loaded PU membrane samples. This indicates that the AgNPs exhibited effective antibacterial activity in both the short and long term. A CCK-8 assay was used in an *in vitro* cell culture to assess the potential toxicity of PTX-loaded membranes on CT26 colorectal cancer cells on Days 1, 3 and 5. The cellular response of the drug-loaded membrane only slightly decreased cell viability after the first day of incubation, and on Days 3 and 5 of incubation the cytotoxicity was significantly higher than that of pure PU, suggesting the potential efficacy of PTX in inhibiting tumor growth. Using a UV spectrophotometer, *in vitro* drug release was evaluated in PBS. After 24 h, the control sample released ∼40% of the drug compared to 20% for the loaded membrane; after 28 days, the control sample released more than 70% of the drug, whereas the loaded membrane only released 57%, this shows that the outermost layer serves the role of sustaining the release profile and inhibiting the initial burst release. Therefore, the coating has slow release and antibacterial effects of anticancer pharmaceuticals and offers long-term patency and therapeutic potential to permit local medication delivery while preventing bacterial adhesion and biofilm formation.

Although the introduction of multiple biological materials may affect the function of these substances [110]. The composite structural design of functional coatings has substantially improved the ability of biliary stents to cope with a range of complex problems, as reflected in many recent research results, and this remains an important area of future research.

## Current challenges and limits

The process of establishing functional coatings should seek to improve drug-loading efficiency and reduce drug waste, which would provide both a guarantee of the effective concentration of the drug and a reduction in cost in practical applications. This should also take into account control of the drug release rate to prevent losses, such as premature or burst release of the drug. Drugs detached from the coating need to maintain a certain concentration to play an effective role, but at the same time bring the possibility of potential toxic side effects on cells and organs. Therefore, keeping the drug release concentration within an effective and safe interval, i.e. balancing the drug concentration between effectiveness and safety, is a key issue in the application of coating technology in biliary stents.

And then, various functional coatings have different application scenarios and face some problems that need special consideration. For example, the hydrophilicity of commonly used antitumor active substances should be controlled within a reasonable range through structural design, and be restricted or optimized according to specific conditions to achieve the best drug release effect. One of the design goals of antitumor coatings is to achieve high concentration local drug administration and targeted drug delivery of tumors. Therefore, it is necessary to research the comparison between the actual effect of the drug-carrying coating and the traditional drug-delivery mode. Another example is that the stone-dissolving coating can effectively dissolve gallstones, and it is also worth considering whether the dissolution process will cause problems, such as blockage in the bile duct or other parts of the bile duct due to the local dissolution of large pieces, which will determine whether it is necessary to control its uniform dissolution.

In addition, biliary stents loaded with functional coatings will eventually need to function in the biliary environment of the human body. The biliary tract is a vast and complex environment; the high fluidity of bile and the dynamic changes in its composition and pH value are all unfavorable factors that may affect the functioning of drugs, so there is no substitute for the necessary human experimentation. The vast majority of current studies are based on cell or animal experiments and the selection of samples in the very few human experiments need to be improved and they have not yet produced sufficiently convincing human research data.

## Perspectives

In recent years, metallic biomaterials, such as stainless steel, cobalt-based alloys, titanium and titanium alloys, zirconium and alloys, have been found to be better than plain metallic biomaterials in terms of biocompatibility and biosafety [111, 112], which provides new opportunities for the development of biliary stent. Coating technology is one of the effective means to improve the performance of the stent, and multi-functional coating may be an important development direction of biliary stent surface coating in the future. In addition, coating technology has the potential to bring solutions to problems in practical applications of biliary stents, such as friction during stent movement.

### Lubrication and antistick coating

The high levels of friction and adhesion present on the surface of many stents can provide possible safety risks during the implantation process and in situations requiring stent removal. In practical applications, lubrication and antistick coating have become increasingly crucial. Currently, there is limited study on the utilization of biliary stent coating. Xu *et al.* [113] presented an excellent slippery surface for biliary stent tips by employing inverse opal colloidal crystals hydrogels. The inverse opal structure provides sufficient room for liquid absorption, such as lubricants and drugs. The slippery liquid-infused porous surfaces (SLIPS) endow the slippery surface with antiadhesion and antibacterial properties. The result shows that SLIPS biliary stent tips have excellent sliding performance. It will most likely reduce biliary tract injury after implantation.

The lubricating and antisticking coating is predicted to aid the stent confront less resistance during movement, which has substantial practical implications for reducing the difficulties and risk of surgery, as well as irritation and injury to the bile duct wall. It is anticipated to be another future direction for biliary stent surface coating technology development.

The above studies employ the notion of bionics to carry out creative coating design, and it also provides us with some recommendations. Many innovative concepts and procedures in various medical stent publications will help to advance coating technology.

### Biomimetic materials

**Figure 9.**
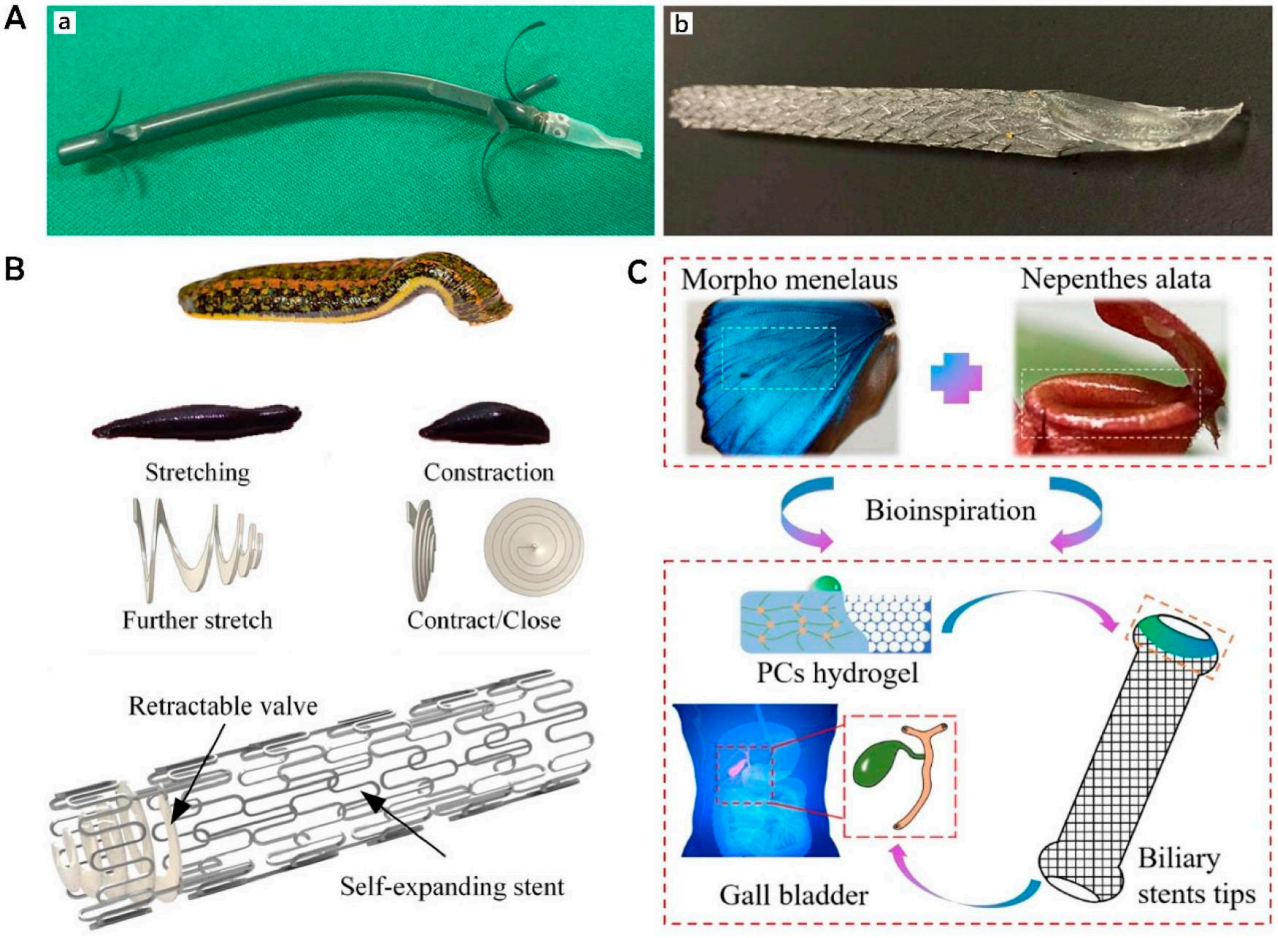
The application of bionic concept in biliary stent. (**A**) (a) Duckbill type antireflux plastic biliary stent. Reproduced from Yuan *et al*. [115] with permission from Copyright 2019 Baishideng Publishing Group Inc. (b) Duckbill type antireflux metal biliary stent. Reproduced with permission from Kobori *et al*.[116]. (**B**) biliary stent with retractable bionic flap in leech form; reproduced with permission from Su *et al* [117]. (**C**) Schemes of biliary stent tips inspired by the combination of multiple organisms. Reproduced from Xu *et al*. [113] with permission from Copyright 2022 Elsevier B.V.

Biomimetics is ‘the practice of designing materials, processes, or products that are inspired by living organisms or the relationships and systems formed by living organisms’ [114]. As an innovative design concept, it draws inspiration from naturally occurring organisms, to try to overcome problems that are difficult to solve in traditional ways. Yuan *et al.* [115] and Kobori *et al.* [116] reported the plastic biliary stent with a duckbill-shaped antireflux valve and the metal biliary stent with a duckbill-shaped antireflux valve, respectively (Figure 9A), both biomimetic stents made of different materials have shown good safety and efficacy when applied to humans. Su *et al.* [117] proposed a new type of antireflux biliary stent with retractable bionic valve based on the characteristics of accordion movement in annelids, such as leech (Figure 9B). The 2D equivalent fluid-structure interaction model based on the axial section and experimental measurements show that the new stent has good antireflux performance, which can effectively prevent duodenal bile reflux and allow bile to pass smoothly, thus reducing cholangitis and increasing patency. Inspired by the super-lubricated surface of *Nepenthes alata* and the photonic crystals microstructure on the wings of *Morpho menelaus*, Xu *et al.* [113] presented the multi-functional SLIPS on the tips of biliary stents, which exhibit adjustable structural color. This technique not only hinders the adherence of proteins and the creation of biofilms, but also possesses the ability to deliver drugs and exhibit antibacterial qualities. As shown in Figure 9C. Organisms in nature provide a wealth of inspiration for people. We can speculate that biomimetic concepts will play an important role in the development of biliary stents.

### 4D printing

The main advantage for resorting to 3DP is represented by the possibility of easily obtaining complex shapes, the products have three dimensions: length, width and height [118]. 4D printing (4DP) relate to the fourth dimension ‘time’, it would involve printing of single- and/or multimaterial objects capable of changing their configuration over time, thus going beyond the concept of 3DP. 4DP items being no longer static objects [119], it not only inherits all merits of 3DP but also allows the static objects created by 3DP to intentionally change their shape, property or functionality over time when exposed to specific external stimuli (e.g. heat, light, water and pH), endowing the printed objects with new features [120]. Interestingly, 4DP is often closely associated with biomimetic materials, e.g. Nature reported on a composite hydrogel [121] that was inspired by a plant system and made using 4DP technology.

The bionic concept and 4DP technology will help the biliary stent to achieve a leap forward development.

## Conclusions

Several different functional coatings discussed in this article showed overall positive results. Despite the research on functional coatings of biliary stents is currently in the early stage, and there are not many relevant studies. We can still draw the following conclusions:

1. The combination of various functional coatings with biliary stents has demonstrated satisfactory efficacy and safety overall; however, the actual effect of the coating was influenced by many factors, including the nature of the loaded material itself, the structural design and preparation method of the coating, the dynamic changes of the stent’s service environment and the stent’s implantation time, among others.
2. Because the human biliary tract environment is dynamic and complex, the *in vitro* simulated environment (artificial bile, human bile *in vitro* perfusion system) and the internal environment of small animals (rats, rabbits and dogs) cannot restore the final service environment of biliary tract stents, and human experiments are irreplaceable, large-scale clinical trials are required.
3. The application of coating technology significantly enhances the performance of the stent. The multi-functional coating on the surface of the biliary stent serves a comprehensive purpose in various complex scenarios. The coating, which possesses lubricating and antisticking properties, holds significant practical importance in the actual use of the biliary stent. These could potentially serve as the guiding principles for the advancement of coating technology in the future.
4. Science, technology and idea innovation will benefit medical materials and give more flexible and efficient solutions for biliary stents, particularly coating technology, bionic concept and 4DP technology.

While the combination of biliary stents with functional coatings is difficult and yet some distance from real-world human applications, the promising prospects it presents are incomparable and worth anticipating.
